# Characterizing phenotype variants of *Cercosporidium personatum*, causal agent of peanut late leaf spot disease, their morphology, genetics and metabolites

**DOI:** 10.1038/s41598-025-85953-9

**Published:** 2025-01-09

**Authors:** Renee S. Arias, Emily G. Cantonwine, Valerie A. Orner, Travis E. Walk, Alicia N. Massa, Jane E. Stewart, John T. Dobbs, Atalya Manchester, Pirada S. Higbee, Marshall C. Lamb, Victor S. Sobolev

**Affiliations:** 1 USDA-ARS National Peanut Research Laboratory, 1011 Forrester Dr. S.E, 39842 Dawson, GA USA; 2 Valdosta State University, 1500 N. Patterson St, Valdosta, GA 31698 USA; 3 Department of Agricultural Biology, Colorado State University, 301 University Ave, Fort Collins, CO USA

**Keywords:** Groundnut, Genetic variants, Morphotypes, Secondary metabolites, Biochemistry, Genetics, Microbiology, Molecular biology, Plant sciences

## Abstract

*Cercosporidium personatum* (CP) causes peanut late leaf spot (LLS) disease with 70% yield losses unless controlled by fungicides. CP grows slowly in culture, exhibiting variable phenotypes. To explain those variations, we analyzed the morphology, genomes, transcriptomes and chemical composition of three morphotypes, herein called RED, TAN, and BROWN. We characterized, for the first time in CP, anthraquinone (AQ) precursors of dothistromin (DOT), including averantin, averufin, norsolorinic acid, versicolorin B, versicolorin A, nidurufin and averufanin. BROWN had the highest AQ and melanin (15 mg/g DW) contents. RED had the highest ergosterol (855 µM FW) and chitin (beta-glucans, 4% DW) contents. RED and TAN had higher resistance to xenobiotics (*p* ≤ 1.0E-3), including chlorothalonil, tebuconazole and caffeine, compared to CP NRRL 64463. In RED, TAN, and BROWN, rates of single nucleotide polymorphisms (SNP) (1.4–1.7 nt/kb) and amino acid changes (3k-4k) were higher than in NRRL 64463. Differential gene expression (*p* ≤ 1.0E-5) was observed in 47 pathogenicity/virulence genes, 41 carbohydrate-active enzymes (CAZymes), and 23 pigment/mycotoxin biosynthesis genes. We describe the MAT1 locus, and a method to evaluate CP-xenobiotic resistance in 5 days. Chemical profiles indicate each CP morphotype could trigger different immune response in plants, probably hindering development of durable LLS resistance.

**Supplementary Information:**

The online version contains supplementary material available at 10.1038/s41598-025-85953-9.

## Introduction

Slow growth and peculiar morphologies in culture have hindered the study of the fungus *Cercosporidium personatum* Berk. & Curtis (CP) (Syn. *Nothopassalora personata* (Berk. & Curtis) U. Braun, C. Nakash., Videira & Crous), causal agent of late leafspot (LLS) in peanut (*Arachis hypogaea* L.)^1^. LLS together with early leafspot disease caused by *Cercospora arachidicola* Hori can result in yield losses of up to 70% if not controlled^2^. Management of these diseases costs $40 million a year to peanut growers in the State of Georgia, U.S.A., who despite frequent fungicide applications experience losses of $12 million to leafspot^3^. CP can be considered hemibiotroph/biotroph, as its close relative *Cladosporium fulvum*^4^.

At present, effective control of peanut leaf spot disease in peanut is normally treated with multiple fungicide combinations that include chlorothalonil (Bravo), prothioconazole + tebuconazole (Provost), pydiflumetofen (Miravis), and azoxystrobin (Elatus), Virginia Tech, Nov 2020. In the early 1990s, chlorothalonil was used at rates of 1.2 kg ai/ha to control late leaf spot, though the dose could be lowered to 0.42 kg ai/ha when combined with cyproconazole^5^. Later, in 2008, it was shown that prothioconazole at 0.18–0.20 kg ai/ha was more effective than tebuconazole 0.23 kg ai/ha in similar regimes of application, and these were more effective than the standard of chlorothalonil 1.2 kg ai/ha^6^. All these fungicides are registered for their use on peanuts, and are currently applied in various combinations and concentrations.

A single spore of CP grown in vitro can take six months to form a 5-mm diameter colony which over time displays color and texture variation^7,8^; spores being multicellular, further confound the work with CP. Some tools recently developed will help advance CP research. The first genome and transcriptome of CP isolate NRRL 64463 have been sequenced and annotated^1^, and image analysis was proven effective to quantify CP growth (https://imagej.net/ij/, National Institutes of Health, Bethesda, MD)^9^, .

Plants have sophisticated sensory systems that activate immune responses when detecting either molecules that are not part of the plant (non self), such as pathogen- or microbe-associated molecular patterns (PAMPs, or MAMPs), or molecules that belong to the plant but are altered, damage-associated molecular patterns (DAMPs)^10^. Examples of effectors recognized by plants are β-glucans, chitin, ergosterol^2^, and melanin^11^; whereas the DAMP response is triggered by products of cell-wall degradation^2,10^, and DNA damage caused by genotoxic agents^12^. Plants can distinguish self from non-self extracellular or extranuclear DNA with high taxonomic specificity, and trigger levels of DAMP response based on DNA length accordingly^13^.

Overcoming plant resistance for phytopathogenic fungi is a matter of time and reproductive biology of the fungus^14^. Sexual reproduction, mutation rate, and high genetic diversity all increase the potential of the pathogen to overcome plant resistance^14^. In Ascomycetes, sexual reproduction is controlled by the mating-type locus (MAT1) which has two idiomorphs, one encoding an alpha-box motif protein (MAT1-1), and the other a High Mobility Group (HMG) (MAT1-2)^15^. We had reported partial information of the CP MAT1 locus^16^, however, detailed annotation of both idiomorphs, crucial information to perform population genetic studies, is not yet available.

Here we analyzed the morphology, genetics, and chemical composition of four different phenotypes of CP isolates to begin understanding the plant offense capability of this pathogen, and the challenge of developing peanut germplasm with LLS resistance.

## Results

### Morphological characterization and growth rate

**Fig. 1:**
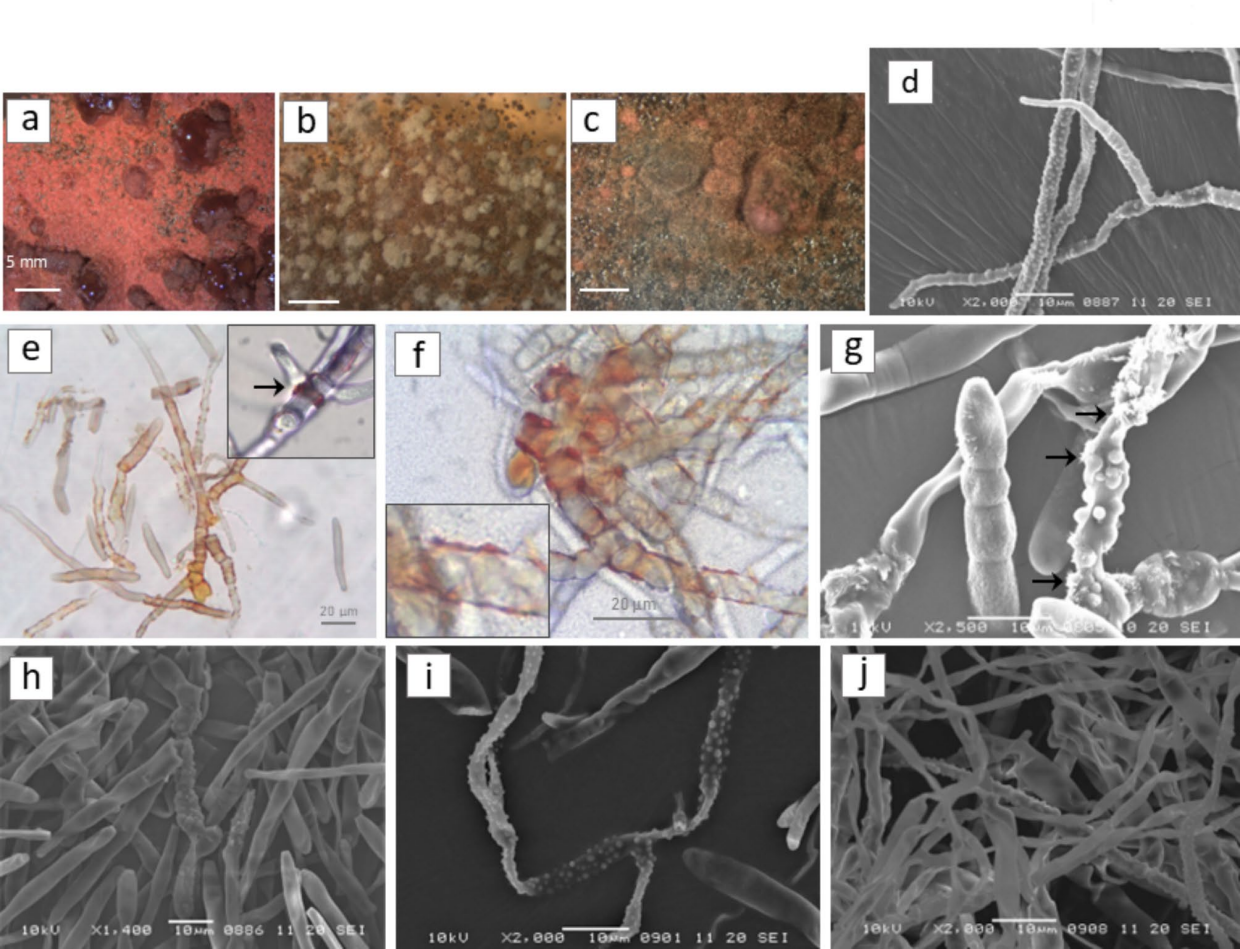
Morphology of three colored CP isolates. **a**, **b**, **c**: stereoscope view 2 x magnification of NPR22 (RED), NPT22 (TAN) and NPO22 (BROWN), respectively. **d**: scanning electron microscopy (SEM) 1000X showing verrucose hyphae in 10-day PDA culture of NRRL 64463. **e**, **f**,** g**: 10-day PDA culture NPR22, left to right, light microscope 400X highlighting crystals, 1000X highlighting pigment droplets, scanning electron microscopy (SEM) showing verrucose hyphae and crystals; → indicates crystals. **h**, **i**,** j**: left to right, SEM showing verrucose structures on 9-day PDA cultures of NPO22, NPR22, and NPT22. Images **a** - **c** were generated by Dr. R. Arias, figures **d** – **j** were generated by Dr. E. Cantonwine.

The three colored-CP isolates are available at USDA-ARS-NRRL collection, Peoria, IL, accessions NRRL 64627 for red morphotype (RED), Fig. 1a, NRRL 64628 tan morphotype (TAN), Fig. 1b, and NRRL 64629 brown morphotype (BROWN), Fig. 1c. Conidia production was widespread after 9-day incubation for BROWN and RED, while sporulation was less abundant and took 2–3 weeks in TAN and NRRL 64463. Orange, extracellular pigments were observed in BROWN and RED, with pigments most frequently occurring as crystals attached to the hyphae in BROWN, and as liquid droplets or crystals in the RED isolate. Pigments were not observed in association with hyphae in TAN or NRRL 64463. RED, TAN and NRRL 64463 developed some verrucose hyphae. Standard hyphal widths were 5 μm, though verrucose hyphae were ≤ 3.5 μm, mean 2.4 μm, and the bump densities were high, with mean bump diameters at 1 μm, Fig. 1d-e-f-g-h-i-j. The verrucose structures observed in BROWN had close association with conidia and shape similar to conidiophores rather than hyphae. RED had a moist, rubbery appearance and flexible consistency, Fig. 1a, compared to the more rigid and dry structure of BROWN and TAN, Fig. 1bc.

### Genome sequencing and analysis of variants

**Fig. 2:**
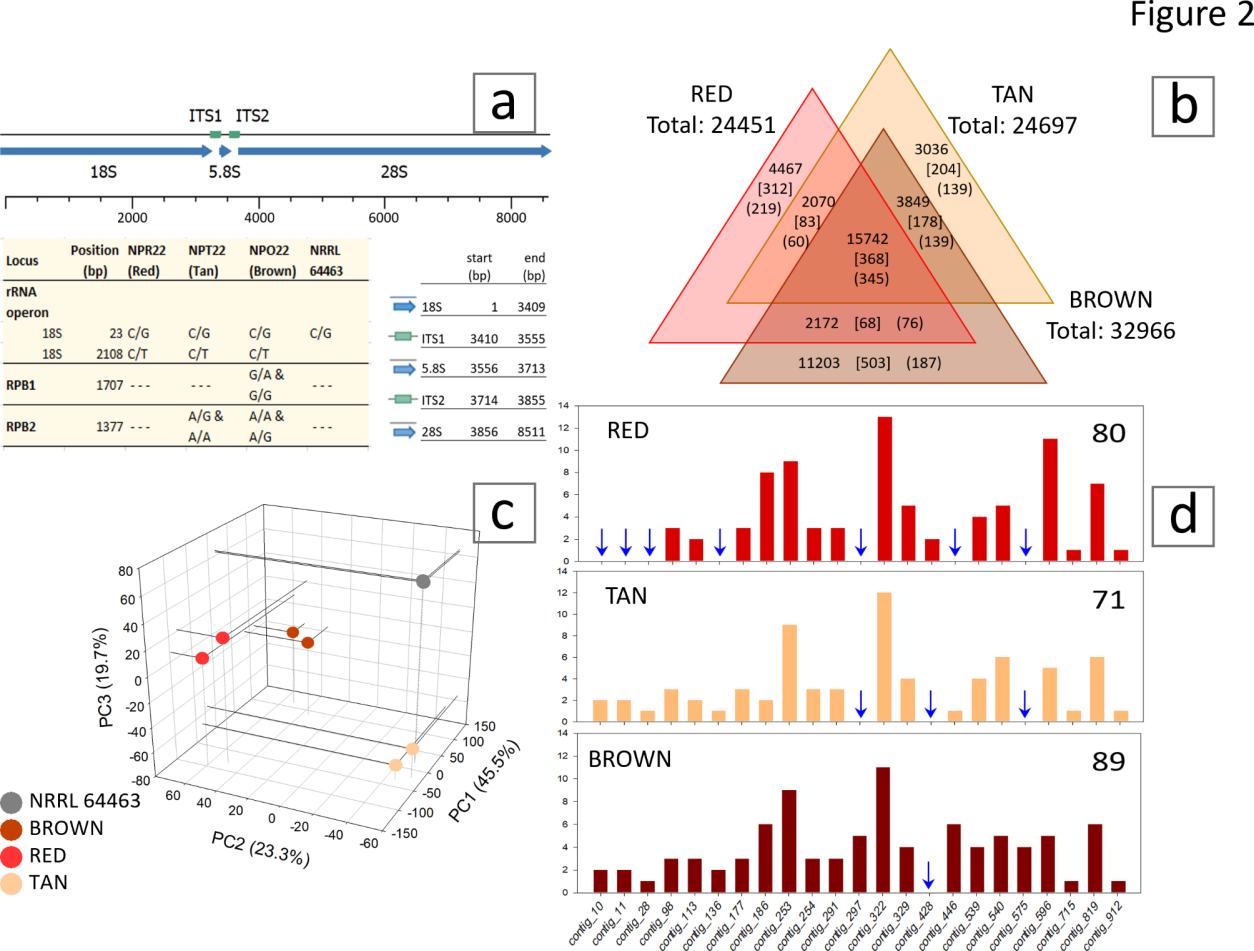
Genomic variants and RNA sequencing summary of three color *Cercosporidium personatum* (CP) isolates. **a**: Annotation of CP ribosomal RNA operon (rRNA), and nucleotide variants in rRNA, largest and second largest subunits of RNA Polymerase II (RPB1 and RPB2). **b**: Genomic variants in three color CP isolates with ≥ 35% frequency not detected in the reference CP genome NRRL 64463, top number: total single- and multi-nucleotide polymorphisms (SNP, MNP), [ ]: number of insertions, ( ): number of deletions. **c**: Principal Component Analysis (PCA) of eight RNA sequencing libraries. **d**: Number of predicted amino acid changes (AAC) on secondary metabolite biosynthesis gene clusters detected by antiSMASH 5.0 reported in 10.7910/DVN/RMBQ0E (Arias et al. 2023), ↓: no AAC predicted.

The raw reads of the genomic libraries of RED, TAN and BROWN, were uploaded to NCBI-GenBank, SRA accessions SRR24707293, SRR24718870 and SRR24718913, respectively, Supplementary Table 1. Mapping genomic sequencing reads to conserved genes: ribosomal RNA operon, RNA-polymerase II largest subunit (RPB1) and RNA-polymerase II second largest subunit (RPB2), showed that color-CP isolates had 99.95% identity to the reference genome NRRL 64463. Nucleotide changes in rRNA operon, RPB1 and RPB2 were 0.23, 0.36 and 0.45 nt/1000 nt. One SNP variant in the rRNA operon was observed in all three color-CP isolates and not present in NRRL 64463. Two SNP variants were detected, one on RPB1 and one on RPB2, Fig. 2a, not present in the published reads of NRRL 64463, Fig. 2a.

Mapping colored-CP genome sequencing reads to the 1061 contigs of NRRL 64463 genome resulted in an average coverage of 140 X, 61 X, and 68 X, for RED, TAN and BROWN CP isolates, respectively. Genome-wide variants using 1% cutoff in all four isolates showed most variants had frequencies higher than 90% or lower than 10%, Supplementary Fig. 2. Using 35% cutoff, most variants were single and multi-nucleotide polymorphisms (SNP, MNP), accounting for 92% in TAN, 91% in RED, and 88% in BROWN. Variants of heterozygous loci (2 or more nt changes) were 69% in TAN and RED, and 80% in BROWN. Genomic variants observed with a ≥ 35% cutoff in the reference genome NRRL 64463 were subtracted from variants of the same percentage cutoff in RED, TAN and BROWN, Supplementary Table 2. The resulting variants were largest in BROWN (32,966), from which 16,455 were shared by all three colored-CP isolates, Fig. 2b. Despite the abundance of variants, all three colored-CP isolates had 99.9% identity to the CP reference genome, confirming their taxonomic identification. Mapping of reads to CBS 151044 chromosomes indicated parts of the 5.7 Mbp-chromosome 6 were missing in the isolates as follows: 26% (1.5 Mbp), 16% (910 kb), 14% (803 kb) and 7% (386 kb) in NRRL 64463, TAN, RED and BROWN, respectively.

### RNA sequencing (RNAseq) and secondary metabolites

#### RNAseq

mRNAs from two biological replicates of 12-day old cultures of RED, BROWN, TAN and NRRL 64463, grown with photoperiod 16/8 h light/darkness were sequenced. A list of sequencing reads and accession numbers at NCBI are in Supplementary Table 3. A principal component analysis (PCA) performed to visualize a summary and quality of the results^17^ showed congruence between replicate samples, and a 92.5% of the variation being explained by the first four dimensions, Fig. 2c. Overall, 5,201 (48%) of the 10,781 CP genes were differentially expressed on the four CP isolates based on a Bonferroni corrected probability *p* ≤ 1.0E-5, 60 of those genes had ≥ 100-fold change. After mapping reads to secondary metabolite gene clusters, predicted amino acid changes (AAC) was highest in BROWN and lowest in TAN, Fig. 2d. Based on the annotated reference genome NRRL 64463^1^ we found significant differential expression (*p* ≤ 10^− 5^) of 41 dbCAN identified genes and 47 PHI-BLAST genes. Heat maps of these results displayed clear patterns between isolates and high uniformity between replicates, Supplementary Figs. 3, 4. Also 192 apoplastic or cytoplasmic genes predicted by Effector-P were differentially expressed (*p* < 10^− 5^) with changes between 2 and 8433-fold.

#### Dothistromin/Aflatoxin biosynthesis gene mini-clusters

**Fig. 3:**
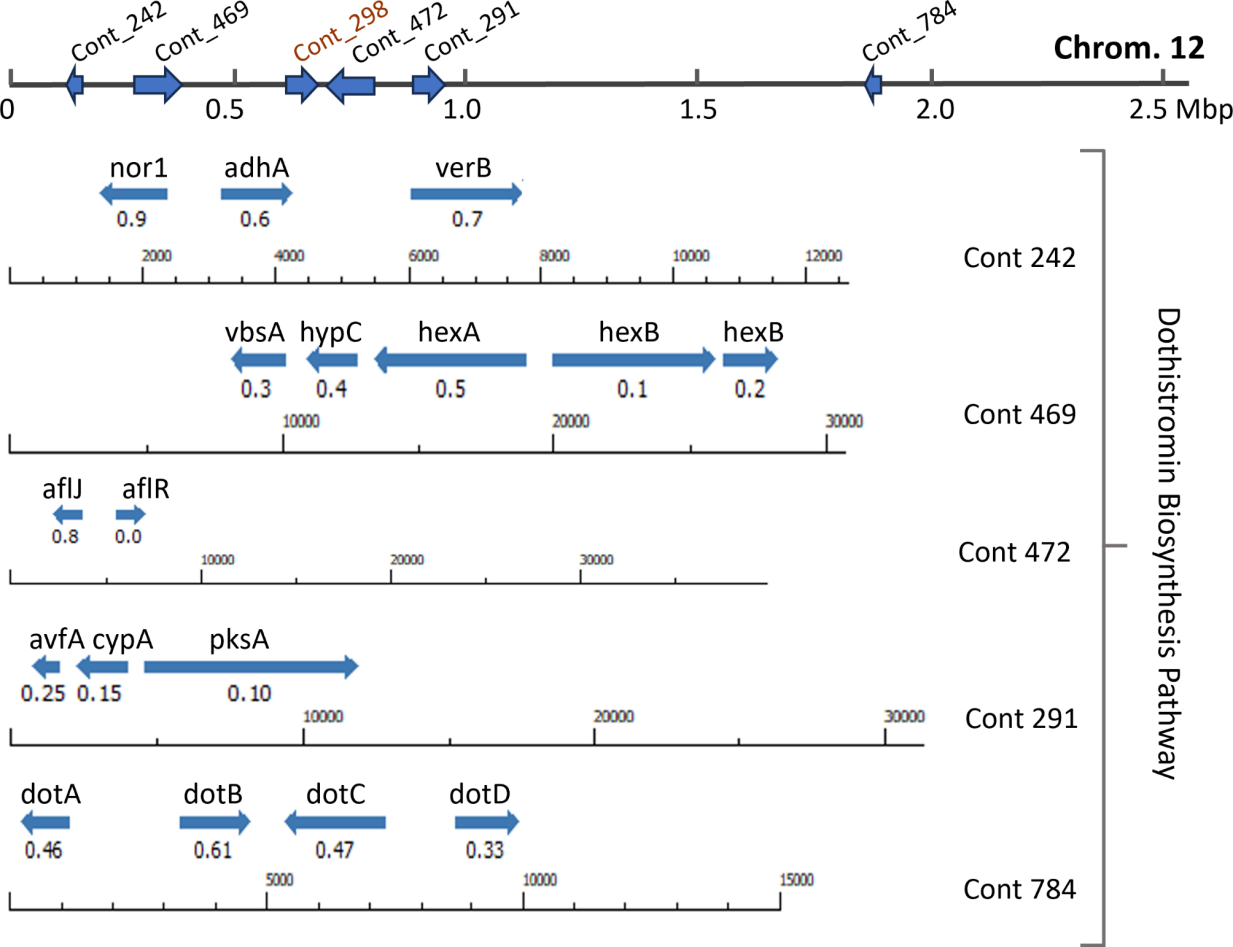
Genes involved in the dothistromin biosynthesis pathway. Scale annotation of mini-clusters in contigs, and contig positions on chromosome 12 of *Cercosporidium personatum*. Contig_298 harbors the mating type (MAT) locus. Contig numbers from Arias et al.. 2023^1^, chromosome reference published in Gonzales et al. 2024^21^, . Mbp: mega basepairs.

**Fig. 4:**
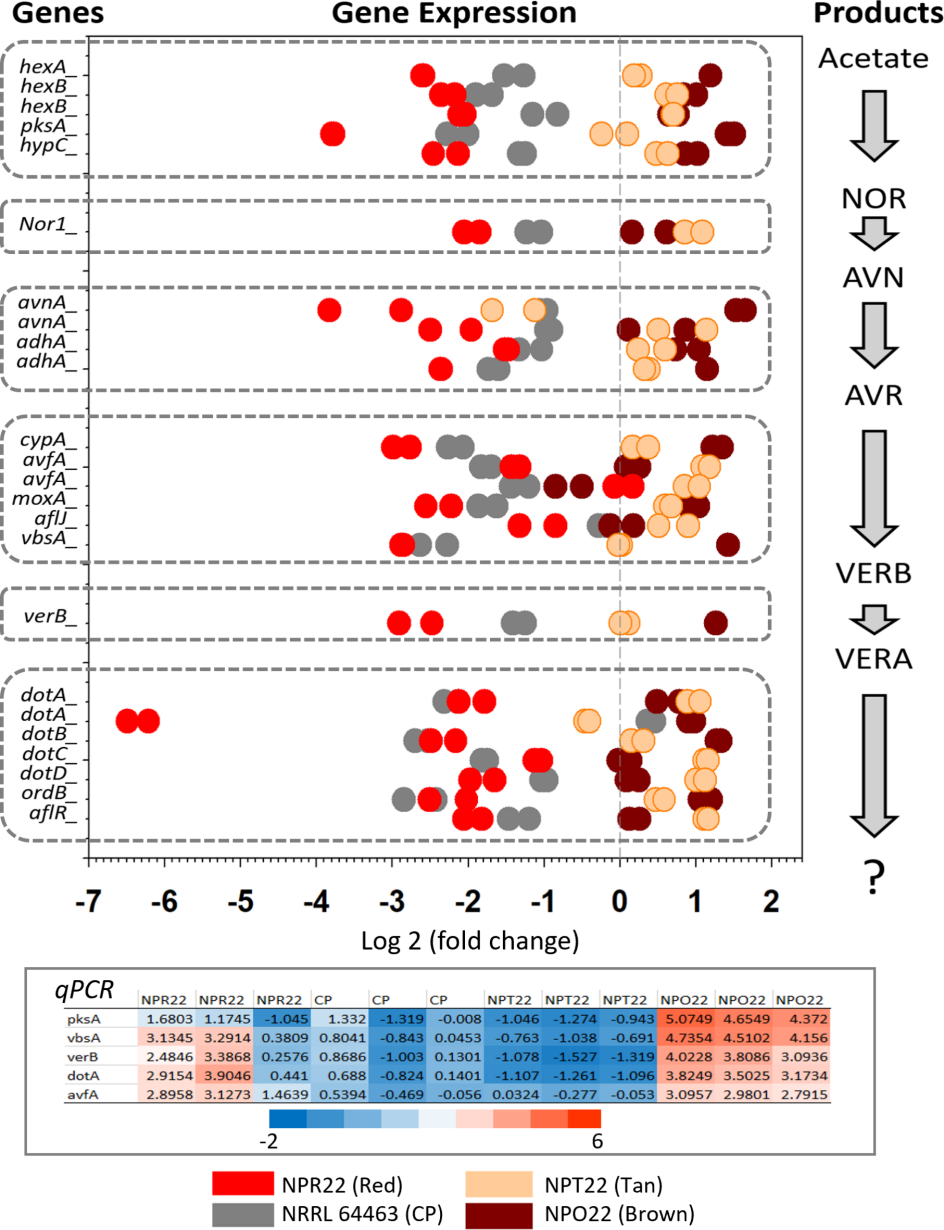
Dothistromin biosynthesis pathway gene expression in four *Cercosporidium personatum* (CP) isolates. **Genes**: orthologous genes from *Fulvia fulva*, *Dothistroma* spp and *Aspergillus* spp. found in CP using the gene models of NRRL 64463 (Arias et al. 2023)^1^. **Gene Expression**: Log_2_ of the fold change, probability level with Bonferroni correction (*p* < 1.0E-5). **Products**: Predicted products for each group of genes and identified in chemical analysis: NOR: norsolorinic acid, AVN: averantin, AVR: averufin, VERB: versicolorin B, VERA: versicolorin A. **qPCR**: results of quantitative Real-Time PCR for some genes in the pathway. **?**: indicates that dothistromin-related compounds are the expected products, though they are presumed present, their identification will require additional work.

Genes functionally confirmed, co-regulated or putatively involved in dothistromin (DOT) or aflatoxin (AF) biosynthesis in *Dothistroma* spp. or *Aspergillus* spp^18–21^. were BLAST to *Cladosporium fulvum*^22^ (NCBI: *JARJJH000000000*) to which CP showed higher homology than to *Dothistroma* spp. The resulting hits were BLAST to the CP reference genome NRRL 64463^1^, and annotated contigs were mapped to the CP chromosomes^23^. All genes identified, including the transcription activator *aflR*, mapped to chromosome 12, except for averantin hydroxylase (*AvnA*) of which two copies were found in chromosomes 4 and 10 (Supplementary Table 4). DOT biosynthesis genes were observed in mini clusters within contigs 469, 242, 291, 472, and 784 of NRRL 64463, their representation at scale is shown in Fig. 3. A complete list of the genes identified in the DOT biosynthesis pathway and their expression levels on CP isolates is shown in Supplementary Fig. 5. Differential expression of transcripts annotated as part of the DOT biosynthesis pathway was evaluated and their predicted gene products later confirmed by LC-MS, Fig. 4. Overall, the highest level of expression of these genes was observed in BROWN and the lowest in RED, Fig. 4. Analysis of gene expression by qPCR of five genes in the DOT pathway showed similar trends compared to the RNAseq data, except in TAN, for which results were inconsistent, Fig. 4.

#### Quantitation of anthraquinones (AQ)

**Fig. 5:**
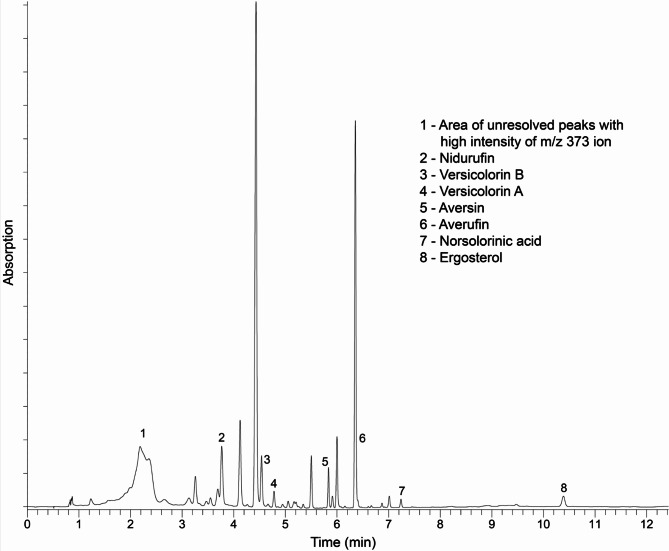
Typical chromatogram observed for *Cercosporidium personatum* (CP) grown for 12 days with photoperiod 16/8 h light/darkness. Image generated by Dr. V. Sobolev.

**Fig. 6:**
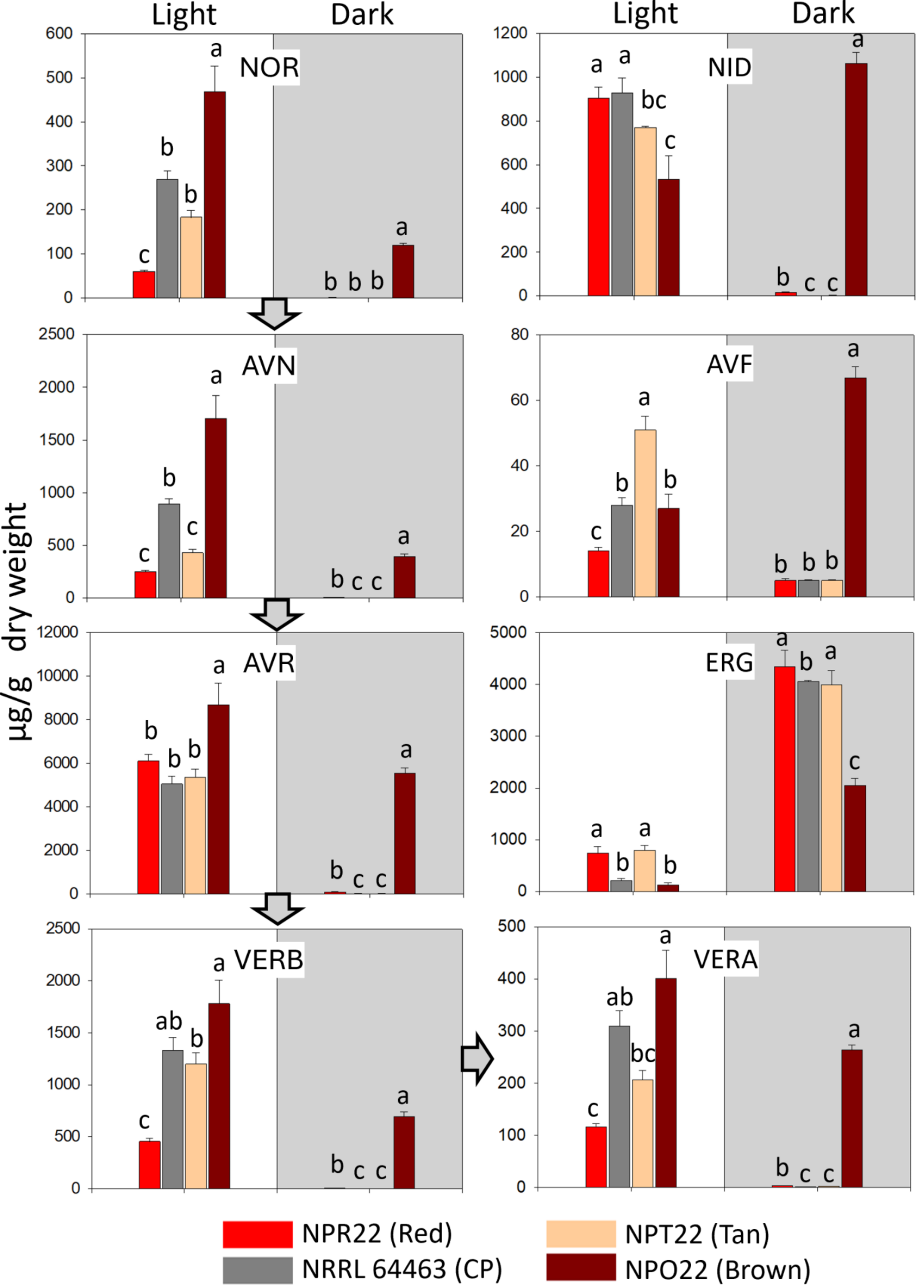
Anthraquinones and ergosterol that were identified and quantitated in four isolates of *Cercosporidium personatum* (CP) using five replicates of each culture grown for 12 days. *Light*: photoperiod 16/8 h light/dark, *Dark*: incubation in the dark (shaded area). Precursors in the dothistromin/aflatoxin pathway are connected by arrows, NOR: norsolorinic acid, AVN: averantin, AVR: averufin, VERB: versicolorin B, VERA: versicolorin A. Compounds related to the pathway but not direct precursors of aflatoxin are AVF: averufanin and NID: nidurufin. Also graphed is the concentration of ergosterol (ERG) that was detected in the analyses. Different letters on the bars of the histograms, within light conditions separately from dark conditions, indicate statistically significant differences, Tukey’s test (*p* < 0.05).

Here we report for the first time in *Cercosporidium personatum* the following AQ being produced: norsolorinic acid (NOR), averantin (AVN), averufin (AVR), averufanin (AVF), nidurufin (NID), versicolorin B (VERB), and versicolorin A (VERA), Fig. 5. These compounds were identified by comparison with authentic standards, and all were detected in the four CP isolates studied. The region marked as **1** in the chromatogram, corresponds to dothistromin-related compounds, Fig. 5. Metabolites that were searched and not found in the samples, based on comparison with authentic standards and within the detection limits of the analysis were: versiconol, versiconol acetate, aversin, ortho-methylsterigmatocystin (OMST), sterigmatocystin (ST), aflatrem, dihydroxyaflavinine, emodin, dihydro ST, dihydro OMST, asparason A, aflatoxins B_1_, B_2_, G_1_, G_2_, M_1_ and B_2a_. The metabolites present in the fungal biomass of the four CP isolates concurred with the levels of expression of DOT-biosynthesis genes. Significantly higher levels of expression and actual concentrations of NOR (468 µg/g Dry Weight, DW), AVN (1704), AVR (8685), VERB (1779) and VERA (401) were present in BROWN, and the lowest concentrations were observed in RED, with NOR (60 µg/g), AVN (249), AVR (6098), VERB (454), VERA (116), Fig. 6. Overall, 7-8-fold more NOR and AVN, and 3-4-fold more VERB and VERA were observed in BROWN compared to RED morphotype, Fig. 6. Two other AQ, Averufanin (AVF) and Nidurufin (NID), were also present in the CP isolates, though these are not precursors in the DOT/AF biosynthesis pathway^23,25^. The highest concentration of AVF was present in TAN (51 µg/g) and the highest NID was present in NRRL 64463 with almost 1 mg/g (929 µg/g DW).

#### Ergosterol

**Fig. 7:**
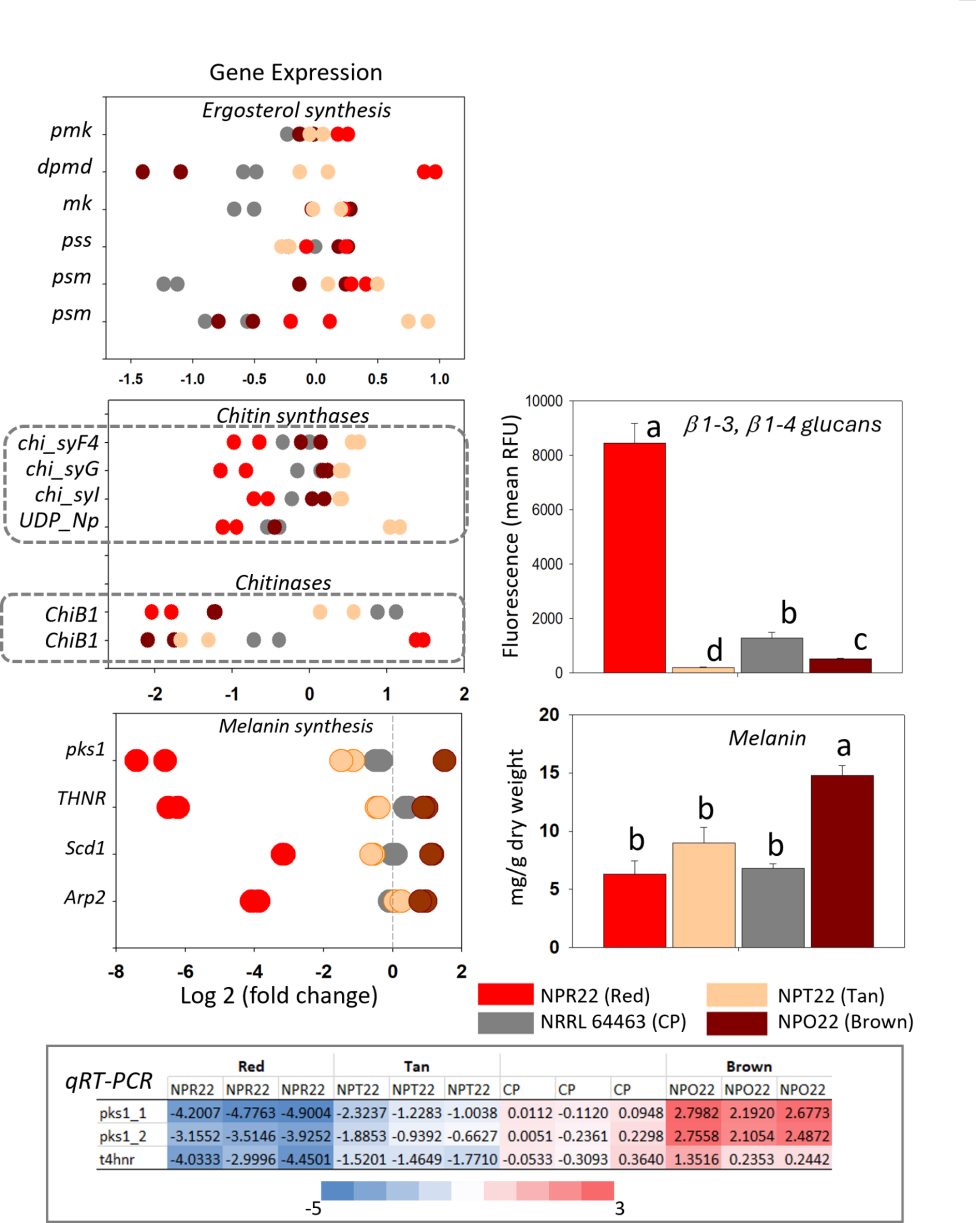
Differential gene expression of ergosterol biosynthesis genes, chitin-synthases, chitinases, and melanin biosynthesis genes in four *Cercosporidium personatum* (CP) isolates. Orthologous genes involved in the biosynthesis of these compounds, and found to have significantly different levels of expression using Bonferroni correction *p* < 1.0E-5 are represented. Quantification of β-[1,3] and β-[1,4] glucans and melanin are shown in histograms where different letters on the bars indicate statistically significant differences, Tukey’s test (*p* < 0.05). qRT-PCR: quantitative real-time PCR for two genes involved in melanin biosynthesis, CP: reference isolate NRRL 64463.

LC-MS analysis detected ergosterol. In cultures grown 12 days with 16/8 h photoperiod and 26 °C, significantly higher ergosterol content (*p* < 0.01) was observed in RED and TAN (743, 792 µg/g DW, respectively), Fig. 6. Concurrently, levels of expression of six ergosterol biosynthesis genes showed correlation with the abundance of ergosterol under photoperiod, Fig. 7. Isolates BROWN and CP had the lowest ergosterol content when grown in photoperiod (100 and 200 µg/g), though showed an ~ 18-fold increase (2000 and 4000 µg/g, respectively) when grown in darkness, Fig. 6. RED grown in darkness reached 4341 µg/g.

#### Chitin, β-[1,3] and β-[1,4] glucans

Most fungal cell walls have a central core of β-[1,3], [1,8] glucan anchored to chitin via a β-[1,4] linkage^26^. Calcofluor white binds to β-[1,3] and β-[1,4] polysaccharides of chitin and cellulose and was used here to quantify these compounds in the isolates. The content of β-[1,3] and β-[1,4] glucans in the isolates was 4.320, 0.856, 0.264 and 0.116 mg/100 mg DW, for RED, TAN, BROWN and NRRL 64463, respectively, and the differences were statistically significant (*p* ≤ 1.0E-2), Fig. 7. However, RED had the lowest levels of expression of four chitin synthases explored, and TAN had the highest, whereas two chitinases with differential expression (*p* < 1.0E-5), had opposite levels of expression in RED, Fig. 7. Among differentially expressed carbohydrate active enzymes (dbCAN), 11 genes were highly expressed in RED and had low expression in other isolates, Supplementary Fig. 3.

#### Melanin

Melanin content in the isolates was quantified using published methods^27,28^, after extracting AQ. Some genes involved in melanin-biosynthesis are polyketide synthase 1 (*pks1*)^29^, scytalone dehydratase (*Scd1*)^30^, hydroxynaphthalene reductase-like (*Arp2*), tetra-hydroxynaphthalene reductase (*Thnr*)^31^. These were differentially expressed across the four CP isolates, with highest expression levels observed in BROWN in RNAseq (*p* < 1.0E-5), confirmed by qPCR (*p* ≤ 1.0E-3) and correlated with the highest melanin content present in this isolate, 14.8 mg/g (*p* ≤ 1.0E-3). Up-regulation of *pks1*, *Thnr*, *Arp2* and *Scd1* in BROWN was 372-, 160-, 30- and 21-fold, respectively, whereas RED had the lowest melanin content (6.3 mg/g), and lowest expression of these genes in RNAseq and qPCR, Fig. 7.

### Pathogenicity

All four CP isolates caused typical leafspot lesions in two peanut cultivars, Georgia-06G and AU-NPL17. Based on 24 inoculation sites, 4–50% of the sites presented symptoms in Georgia-06G, and 8–46% in AU-NPL17. The largest number of lesions were observed on different isolates in repeated experiments, thus, there was not sufficient evidence to consider any more virulent than others based on the detached-leaf assay.

### Mating type

We described for the first time the CP MAT1 locus, including MAT1-1 and MAT1-2 genes orthologous to those in *Cladosporium fulvum* Race 5^22,32^. CP contig_298 was located on chromosome 12, flanked by DOT biosynthesis gene mini clusters, Fig. 3. A dot plot of NRRL 64463 contig_298 *vs*. the orthologous region in CBS 151044 showed a gene inversion around position 35 kb, on the MAT + sexual cell fertilization-promoting factor (augustus_masked-contig_298-processed-gene-0.26), labelled as MAT1-2 in the colored CP isolates and NRRL 64463, Supplementary Fig. 6. We found that all three colored CP isolates studied here and the reference isolate NRRL 64463 harbor the MAT1-2 gene, whereas isolates IPAVE 0302^16^ and CBS 151044^23^ harbor the MAT1-1 gene, Supplementary Fig. 7. Reverse primers designed for MAT1-1 and MAT1-2 of *Cladosporium fulvum*, (MAT1-1 P4R 5’-TGTTCGGTGTCGTGATG-3’, MAT1-2 P4R 5’-TCCACGTCGAAGTAGAG-3’)^32^ were both found in CP MAT1 locus, but the forward primers did not match. Hence, we designed forward primers: MAT1-1_CPFw 5’-TCCTGTGGCGTGCCGATCCC-3’, and MAT1-2_CPFw 5’-TACCCACCGACCATGCTATC-3’, that in combination with the reverse primers reported^32^ will amplify a 759 bp and a 997 bp fragments of idiomorphs MAT1-1 and MAT1-2, respectively.

### Response to xenobiotics

**Fig. 8:**
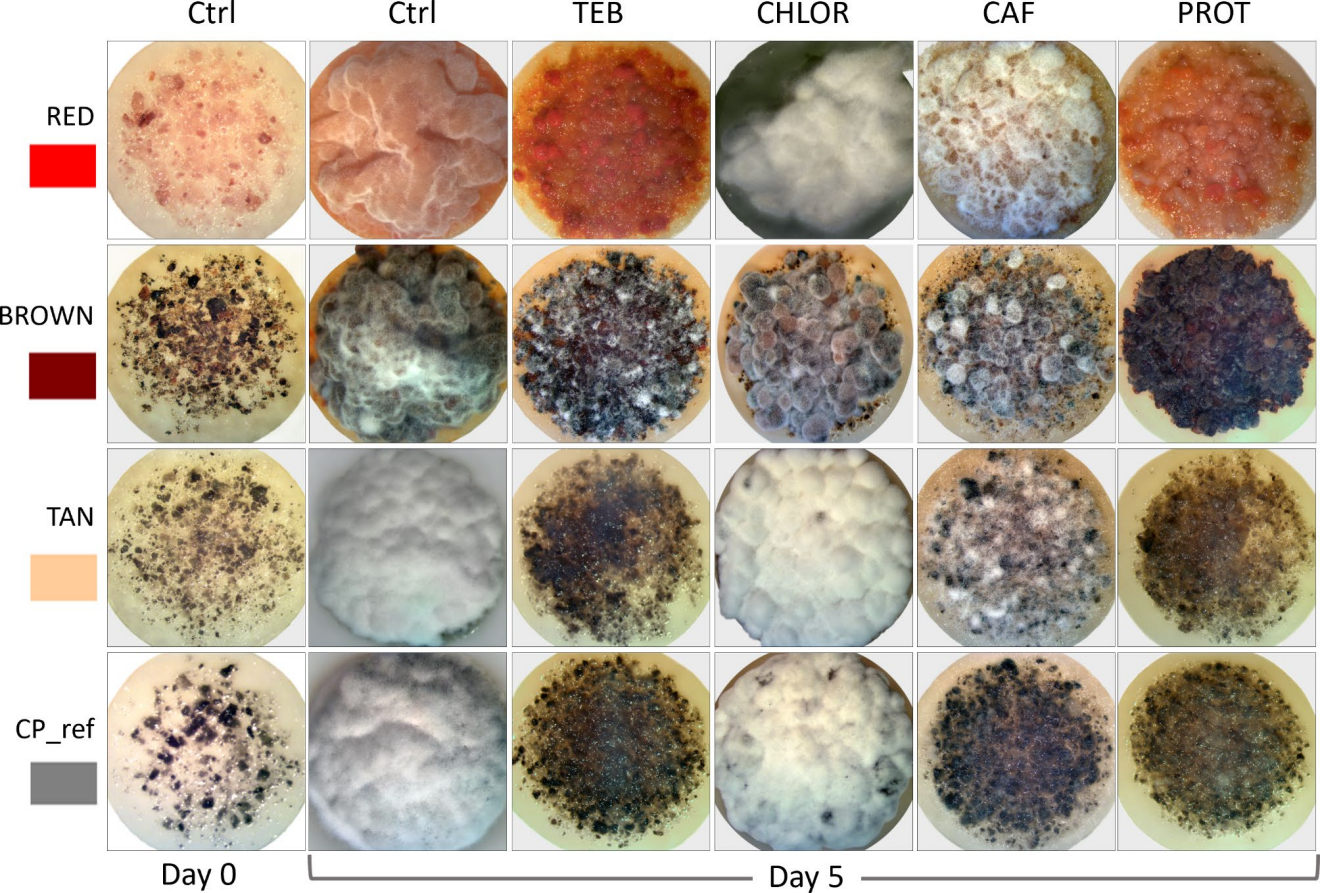
Response of four *Cercosporidium personatum* (CP) isolates to xenobiotics. First and last column correspond to the controls (Ctrl) on potato-dextrose agar medium at 0- and 5-days incubation. PROT, TEB and CHLOR are images obtained after 5 days at 10 mg a.i./L of prothioconazole, tebuconazole and chlorothalonil, respectively. CAF are images after 5 days at 10 mM caffeine. Images were generated by Dr. R. Arias.

Overall, the three color-CP isolates had higher levels of resistance to chlorothalonil (CHLOR), tebuconazole (TEB), and caffeine (CAF) compared to the reference isolate NRRL 64463 (*p* < 0.05), whereas TAN was slightly more susceptible to prothioconazole (PROT) (*p* < 0.05), Supplementary Fig. 8. The recommended inoculum size, 5-day incubation and the use of PVDF membranes were suitable to test CP fungicide resistance, Fig. 8. Average dose of xenobiotics that inhibit growth by 50% (EC_50_)^33^ were calculated for two experiments using AAT Bioquest for four parameters^34^, and were: CHLOR 1.06 mg/L, PROT 1.56 mg/L, TEB 18.22 mg/L, and CAF 7930 mg/L (40.84 mM). Both PROT and TEB are triazole fungicides, and since changes in the sterol 14-α demethylase (cyp51) gene are usually associated to azole-fungicide resistance^35^, we performed BLAST search for this gene in the reference CP transcriptome. Gene sm_ctg_825 − 0.1 corresponded to sterol 14-α demethylase and was up-regulated (*p* < 10E^− 10^) by 23-, 8- and 3-fold in RED, TAN and BROWN, respectively. BLAST search identified 11 glutathione S-transferase (GST) genes (E < 10^− 4^) in the CP transcriptome, nine were differentially expressed (*p* < 10^− 10^), and eight of those nine had highest expression in TAN, up to 20-fold more than other isolates, whereas the lowest expression occurred in NRRL 64463. The level of resistance to CHLOR depicted in the histogram for 3.3 and 10 mg/L, Supplementary Fig. 8, correlated with GST expression levels on CP isolates.

## Discussion

Single spore isolates of *Cercosporidium personatum* (CP) often show irregular morphology in culture^1^. Here we explored some probable causes of variations in four CP isolates to help understand the pathogen/host interaction. We first confirmed all four isolates were a single taxon, as highly conserved genes, RPB1, RPB2, rRNA operon, each showed ≤ 1 nt difference in colored CP when compared to NRRL 64463, Fig. 1a, and genome-wide SNP rates (1.4–1.7 SNP/kb) granted a 99.9% identity, Fig. 2b. In comparison, isolates of the plant pathogen *Puccinia triticina*, had rates of 2.4 SNP/kb^36^, whereas rates of 3.8–5.0 SNP/kb were observed among L-morphotype isolates of *Aspergillus flavus*^37^. In addition, the higher number of genomic variants at > 90% and < 10% frequencies (Supplementary Fig. 2) were consistent with a homokaryotic distribution^38^.

Anthraquinones (AQ) biosynthesis genes and metabolites. CP and *C. arachidicola* (CA) are phylogenetically closely related^1^. Since CA can produce dothistromin (DOT)^39^, a toxin originally described in *Dothistroma septosporum*^19^, we speculated DOT could be present in CP. In *D. septosporum*, DOT biosynthesis genes are organized in mini-clusters^40^, we identified orthologous genes in CP also organized in mini-clusters, Fig. 3. These co-located to chromosome 12 and had several gene duplications in other chromosomes. Such duplications were also reported in *D. septosporum*^19^. Despite grown in the same conditions, each CP isolate showed contrasting patterns of gene expression throughout their genomes, Figs. 4 and 7, Supplementary Figs. 3, 4, and different concentrations of secondary metabolites, Figs. 6 and 7. AQ such as NOR, AVN and AVR^25^, are precursors of the mycotoxins DOT/AF, which share the same pathway between Acetate and VERA^19^, Figs. 4 and 6. With each step of the pathway, the compounds become more toxic. AVF and NID are not directly involved in the biosynthesis of DOT/AF; AVF results from artificial dehydration of hydroxyaverantin (HAVN)^24^, whereas a small fraction of HAVN, in the presence of NADPH or NADP gets converted to NID^25^. NID, AVN and AVR have shown antimicrobial activity against Gram positive bacterial pathogens with minimum inhibitory doses (MIC) of 2.3, 4.4 and 10.0 µg/mL, respectively^41^. Based on fresh weight, our CP isolates, under photoperiod, accumulated 8-, 9-, and 39-fold above MIC values (19, 42 and 394 µg/mL) for AVN, NID and AVR, respectively, Fig. 6. It would be interesting to examine if these compounds affect the plant microbiome and susceptibility to LLS disease.

Genotoxicity, or DNA damage in plants, is usually associated to abiotic stresses^42^. However, pathogen effectors can damage DNA and trigger DNA damage response (DDR), damage repair, and in severe cases lead to apoptosis^43^. Whereas NOR and NID have no genotoxicity, from AVR, VERB to VERA the genotoxicity increases^44,45^. Based on fresh weight, under photoperiod CP isolates produced 27 to 93 µM of VERA. As a comparison, colon cancer cells exposed to 1 µM of VERA experienced differential expression of 18,000 genes, whereas only 869 genes were differentially expressed with 1 µM aflatoxin B_1_ (AFB1); and VERA triggered apoptosis, showing 10-fold more genotoxicity and four-fold more oxidative stress than AFB_1_^46^. Isolate BROWN had the highest accumulation of NOR, AVN, AVR, VERB and VERA, under photoperiod and darkness, Fig. 6. VERB and VERA possess a bis furan moiety which binds to DNA conferring mutagenic activity^21^, the furan ring causes DNA damage and chromosomal aberrations^47^. Exposure of ten types of cancer cells to AVF, NID, and AVR resulted in the following IC_50_, AVF: 11 µM, NID: 40 µM, AVR: 26 µM, in addition AVF caused apoptosis and cell cycle arrest at G1 phase^48^. Based on fresh weight, our CP isolates had AVF concentrations close to IC_50_, both in photoperiod and darkness (1–14 µM), and NID concentrations in photoperiod (108–189 µM) or darkness (0.2–216 µM), 3-5-fold above its IC_50_. Overall, if considering growth in photoperiod, BROWN produced higher concentration of compounds known to be genotoxic (AVR, VERB and VERA), whereas the other three isolates produced more of the compounds known to induce apoptosis (NID, AVF). In terms of plant response, DDR, has been shown to activate salicylic acid accumulation associated with plant defense against biotroph pathogens^49^.

Melanin and β-glucans. Melanins are complex and multi-functional polyphenol biopolymers that can act as antioxidants, confer resistance to radiation and osmotic stress, work as energy storage, and be involved in pathogenesis^11^. There are at least eight types of melanin^50^. They can accumulate on various fungal structures and at different ratios playing different roles in pathogenicity^50^ and can also confer some resistance to antifungal drugs^11^. In our work, BROWN contained 1.48 mg melanin/g DW, 1.6- to 2.3-fold higher than the rest of the isolates, explaining the brown coloration; however, this isolate did not show significant advantage when exposed to four xenobiotic compounds, Supplementary Fig. 8. The CP isolates had between 0.4 and 1.6% DW melanin which is within the common range, 0.4 and 8.0% melanin, in fungal biomass^27^, though we only extracted and quantified insoluble melanin^27,28^. In humans, the photoprotection effect of melanin in skin depends not only on the types of melanin present (eumelanin, pheomelanin) but also their ratios^51^. Thus, further work to characterize melanin in CP would be helpful. It was recently discovered that most enzymes in the 1,8-dihydroxynapthalene (1,8-DHN) melanin pathway are shared with the perylene quinone mycotoxin pathway, e.g. altertoxin (ATX)^52^, thus, this possibility could be explored in CP to better understand its interaction with the peanut plant.

Two interesting observations derive from the quantification of β-[1,3] and β-[1,4] polysaccharides of chitin. First, that β-[1,3]-glucan-chitin make a particularly flexible viscoelastic network^53^, therefore, the rubbery, flexible consistency of the red morphotype, RED, may be the result of its significantly higher content of β-glucans compared to the rest of the isolates. Second, that chitin and chitin-related compounds activate the biosynthesis of jasmonic acid (JA), which is associated with the plant response to necrotroph pathogens^54,55^.

Mating type. This is the first report of MAT1 locus genes and heterothallic nature of *Cercosporidium personatum*. Here we compared three new CP genomes with those previously reported^1,16,23^ and show each harbored only one MAT1 idiomorph, either MAT1-1 or MAT1-2 but not both, therefore indicating these isolates are heterothallic. We had reported CP contig_298 contained MAT1 locus^16^. Here we detailed the structure of MAT1 locus in chromosome 12 flanked by DOT biosynthesis gene mini-clusters, the gene sequences of MAT1-1 and MAT1-2, and primers for their detection in population studies. Following the nomenclature for mating type genes in filamentous ascomycetes^15^, the four CP isolates studied here had the High Mobility Group (HMG) type, MAT1-2 gene (am_ctg298-0.26); whereas CBS 151,044^23^ and IPAVE 0302^16^ had the Alpha Box motif protein (MATα1) or MAT1-1, Supplementary Fig. 7. Equal ratios of mating types in fungal pathogen populations are indication of sexual reproduction and increased prospect of overcoming plant host resistance^56^. Therefore, these primers will be crucial to analyze mating type distribution in the LLS pathogen populations.

Xenobiotics. This is the first report of response to fungicides by individual CP isolates using image analysis. Image analysis was recently developed to evaluate the recalcitrant growth habit of peanut leafspot pathogens CP and CA^9^. To test CP exposure to xenobiotic compounds we standardized inoculum size, introduced the use of PVDF membranes on the medium, and determined suitable incubation conditions that allowed results in 5 days. Normally, evaluating fungicide resistance in individual CP isolates would take 3–6 months, and the impracticality of this is pointed out in the literature^57^. A previous method developed to evaluate fungicide resistance used 15–20 peanut leafspot lesions from the field to obtain results in 14–30 days^57^ by the detached leaf assay^58^. The method we describe is not only completed in 5 days, but it also allowed us to calculate the EC_50_ of fungicides, whereas more accurate estimations can be obtained by increasing the number of isolates.

Multiple applications of fungicides are necessary during the peanut cropping season to control leafspot diseases^59,5^. Among these products are the sterol biosynthesis inhibitors (SBI) TEB and PROT, and the multi-site fungicide of the chloronitrile group CHLOR^6^, the latter being used as the standard when comparing fungicides^60^. In our experiments, expression of eight glutathione S-transferase (GST) genes was highest in TAN and lowest in NRRL 64463, which correlated with their levels of resistance to TEB and CHLOR (ea., 10 mg/L). GST catalyzes the conjugation of the glutathione sulfur atom to xenobiotic compounds resulting in detoxification^61^. The three colored morphotype CP isolates, RED, TAN, BROWN, were collected in 2021, and had higher resistance to TEB, CHLOR, and PROT than NRRL 64463 which was isolated in 2013, Supplementary Fig. 8. This is not surprising since the effectiveness of these fungicides has been reported to decrease over the years^6^. Caffeine was included in the tests because gene sm_ctg_390 − 0.41 which encodes for a caffeine resistance protein, was expressed 2,300-fold higher in TAN compared to the rest of the isolates, though no correlation was observed with its caffeine tolerance.

Ergosterol. Ergosterol (Ergosta-5,7,22-trien-3β-ol; C_28_H_44_O, MW:396.34) is an essential component and the most abundant sterol of fungal cell membranes and has an essential role in membrane stabilization^62^. Within fungal species, ergosterol content is considered rather constant^63^ whether a culture is grown in photoperiod or darkness^64^, hence it is often used to estimate fungal biomass in ecosystems^65,66^. Thus, it was surprising to find that CP isolates grown in the same conditions had statistically significant differences in ergosterol content (*p* < 0.01). RED grown in darkness reached 4.5 mg/g DW ergosterol, similar concentrations have been reported in other fungal pathogens, e.g., between 4 and 6 mg/g DW in *Trichoderma harzianum*, 3.9 mg/g DW in *Botrytis cinerea* and 6.7 mg/g DW in *Fusarium sporotrichioides*^67^. In RED and TAN, the highest ergosterol content in photoperiod (*p* < 0.01) (Fig. 6), correlated with high ergosterol biosynthesis gene expression, mevalonate kinase, diphospho-mevalonate kinase, and putative squalene monooxygenases (Fig. 7), and high resistance to TEB (10 mg/L), Supplementary Fig. 8. These two isolates also showed a moderate ~ 5-fold increase in ergosterol content when grown in darkness, whereas BROWN and NRRL 64463 experience 18-fold ergosterol increase in darkness, Fig. 6. The ergosterol molecule and its biosynthesis genes are targets of important groups of fungicides such as azoles and polyenes, noting that changes in ergosterol abundance affect the level of susceptibility to these drugs^68^. Consequently, the sizable variation in ergosterol content among CP isolates and growing conditions, deserves consideration when assessing fungicide resistance in the LLS pathogen.

Ergosterol is not produced in plants, therefore it is recognized as a “non-self” molecule by the plant immune system^10^. Ergosterol is an effector of the microbe/pathogen-associated molecular pattern (MAMP/PAMP) recognition along with chitin (β-glucans)^55^. Based on fresh weight, some CP morphotypes accumulated 0.5–1 mM ergosterol. It is known that as small as nanomolar concentrations of ergosterol can activate production of salicylic acid (SA) (response to biotrophs)^62^. However, the response to ergosterol also depends on the abundance of squalene, precursor of ergosterol, and on the ergosterol/squalene ratio where the transcription factor, WRKY40, has been shown to positively modulate JA and inactivate SA^69^. Other CP morphotypes produced significantly higher concentration of genotoxic AQ. In general, genotoxicity, DNA damage, is known to activate SA production^49^. Yet, another morphotype accumulated up to 40-fold more chitin than other isolates. In plants, chitin activates the jasmonic acid biosynthesis pathway (response to nectrotrophs)^54^. In summary, different CP morphotypes could facilitate suppression of either JA or SA. Such changes in JA or SA response could render a plant more susceptible to pathogens (here morphotypes) that have a different mode of invasion^70^. We speculate, CP morphotypes could cooperate among themselves to achieve plant host invasion.

## Methods

### Fungal isolation

Color variant isolates of *Cercosporidium personatum* (CP) used in this work were initiated in culture from sporulating late leafspot lesions of peanut plants cv. Georgia-06G, collected in October 2021 at the University of Georgia Coastal Plain Experiment Station, Lang Farm in Tifton, Georgia (Lat.: 31.476099 N; Long.: -83.5248768 W). Lesions were dried at room temperature and stored in glass vials for six months at 4 °C. After five months of incubation, tissues with the original brown morphology, having dark brown, pulvinate stroma with a hard texture (NPO22: BROWN), and those of a tan color morphological variant having similar stroma covered with tan or light brown prostrate hyphae (NPT22: TAN), were excised from the same plate and sub-cultured. A red variant, having red stroma with a smooth and soft texture (NPR22: RED) was obtained from a separate culture. The morphology of BROWN, TAN, RED cultures was characterized. Excised stromatal tissues of the cultures were homogenized separately in 1 ml sterile water using a LabGEN 125 Tissue Homogenizer (Cole Parmer, Vernon Hills, IL) and transferred to fresh potato-dextrose agar (PDA) plates (potato dextrose broth Difco cat. # 254920, with 15 g/L agar Fisher cat. # BP1423), to cultivate morphotype cultures and initiate nonstromatic growth, e.g. filamentous hyphae or conidia, for further study. All these isolates were vouchered with the Valdosta State University (VSC) Fungarium, uploaded to MycoPortal, and submitted to the USDA-NRRL culture collection in Peoria, IL, USA.

### Morphological characterization

Morphological observations were conducted for three isolates of the original brown morphotype, three of the red morphotype, and one tan form. Following the transfer of conidia or filamentous hyphae induced by homogenization to fresh PDA plates, 9-day old cultures were examined at 400X magnification for the presence or absence of conidia, verrucose hyphae, and orange color extracellular pigments present either as droplets near hyphae or as crystallizations on hyphal surfaces. Five fields of view chosen at random were observed for each sample. Scanning electron microscopy (SEM) was conducted using a JEOL 6480 LV scanning electron microscope (JEOL, Tokyo, Japan) to confirm and characterize verrucose hyphae. Tissues for SEM analyses were prepared from sections of 9-day old cultures that had been dried at 46 °C for 24 h. Tissues were affixed to aluminum SEM pegs using double-sided carbon tape and coated with gold/palladium using a Denton Vacuum Desk IV sputter-coater (Moorestown, New Jersey, USA). The presence or absence of verrucose hyphae were observed at 1000X to 3000X. When present, verrucose lobe densities were rated as low, medium, or high, and the diameters of verrucose hyphae and individual lobes were measured.

### DNA extraction and sequencing

The three color-CP isolates, BROWN, RED and TAN, were grown on PDA medium at 26 °C as initial inoculum. Approximately 1 cm^2^ of each culture was separately placed in sterile test tubes containing 2 mL sterile distilled water, ground with a LabGEN 125 Tissue Homogenizer, then, 400 µL of each culture suspension were spread on PDA plates. The plates were incubated at 26 °C ± 0.5 °C 16 h light/8 h dark to induce sporulation. Biomass, including spores and hyphae were harvested after 9, 13 and 18 days for BROWN, RED and TAN, respectively. DNA was extracted from the fungal biomass using DNeasy Plant Mini Kit in a QIAcube robot (both from Qiagen, Redwood City, CA, USA). Genomic DNA libraries were prepared for each color CP isolate using Illumina TruSeq Prep Kit V2 (Illumina, San Diego, CA), and sequenced as paired end (PE) 150 base pairs (bp) on Illumina NovaSeq 6000 System (Illumina, San Diego, CA) at the LC Sciences, Houston, TX.

### Mapping and analysis of genomic variants

Given the various morphologies of the colored CP isolates, we first confirmed their taxonomic identification by mapping the sequencing reads to highly conserved genes commonly used in phylogenetics of Ascomycetes. After removing low quality reads and potential adapters, reads were mapped to 8511 bp ribosomal-RNA operon, 5523 base pairs (bp) of the RNA-polymerase II largest subunit (RPB1) and to 4434 bp of the RNA-polymerase II second largest subunit (RPB2) of the reference genome NRRL 64463. Reads of RED, BROWN, and TAN (this work) and the reads of NRRL 64463 (NCBI: SRR22033838) genome^1^ were then mapped to the 1061 contigs of NRRL 64463, and genetic variants searched using the following parameters: ploidy = 1, minimum count = 2, minimum coverage = 10 (10% of the read coverage at that locus), minimum frequency = 35%. Genome-wide variants (insertion/deletions (InDels)), and single- or multi-nucleotide polymorphisms (SNPs, MNPs) were recorded. Variants were detected using the same parameters and a minimum frequency of 1% cutoff was used to observe allele frequency distributions, and only hyper-allelic variants were computed. All analyses were performed using CLC Genomics Workbench 23.0.4 (Qiagen, Aarhus, Denmark).

### RNA sequencing and data analysis

The three color-CP isolates and the reference culture NRRL 64463 were grown each on 20 plates of PDA medium and incubated at 26 °C ± 0.5 °C with photoperiod 16 h light/8 h dark for 12 days. Their biomasses were collected on separate 50 mL sterile centrifuge tubes and stored at – 80 °C approximately 2–3 weeks until processing. Total RNA was extracted from two biological replicates of each isolate, mRNA libraries were prepared using TruSeq RNA library prep kit v2 (Illumina, San Diego, CA), and sequenced on Illumina NovaSeq 6000 at the University of Minnesota Genomics Center, MN, (https://genomics.umn.edu). RNA sequencing data were normalized using trimmed mean of million (M) values (TMM) method^71^, and Principal Component Analysis (PCA)^72^ was performed to visualize the variation between isolates. Based on the annotated reference genome NRRL 64463, differential expression analysis was performed according to Mortazavi et al.^73^ with parameters set to Bonferroni corrected p-value ≤ 1.0E-5, and Log_2_ of absolute fold change ≥ 2. Heat maps were created for various groups of genes of interest including pathogenicity/virulence genes identified by pattern-hit initiated BLAST (PHI-BLAST)^74^, carbohydrate active enzymes (dbCAN2)^75^, apoplastic and cytoplasmic effectors according to Effector_P^76^, and dothistromin/melanin/chitin/ergosterol biosynthesis related genes^18,19,22,77^. In addition, using previously identified secondary metabolite gene clusters^1^, we searched the genomes of color-CP isolates for predicted potential amino acid changes. All analyses were performed using CLC Genomics Workbench 23.0.2.

### Quantitative real-time-PCR of pigment biosynthesis genes

Using the genome annotation of NRRL 64463^1^, both genes and transcripts of five genes in the dothistromin-biosynthesis pathway (*pksA*, *vbsA*, *dotA*, verb, *avfA*) and two in the melanin-biosynthesis pathway (*pks1*, *t4hnr*) were selected for quantitative Real-Time PCR (qPCR). Genes and transcripts were aligned and primers were designed to span introns using Clone Manager v.11 (Sci. Ed. Software LLC, Westminster, CO). The three color-CP isolates and the reference NRRL 64463 were grown on multiple PDA medium plates and incubated at 26 °C ± 0.5 °C with photoperiod 16 h light/8 h dark for 12 days. Fungal biomass was collected on separate 50 mL sterile centrifuge tubes, stored at -80 °C, and total RNA was extracted within one week after collection. Five cDNA reaction synthesis were prepared using 8 µg of total RNA from each of three biological replicates with a combination of random hexamers and oligo-dT. Superscript III First Strand Synthesis Super Mix (Cat. # 18080400) (Invitrogen by Thermo Fisher Scientific, Waltham, MA) was used according to manufacturer’s instructions. qPCR was performed on an Applied Biosystems QuantStudio 7 Pro instrument (Thermo Fisher Scientific, Waltham, MA), using RT2-SYBR Green ROX qPCR Mastermix (Cat. # 330523) (QIAGEN, Germantown, MD). The qPCR was performed in 24 µL reactions with 5 µL of a ¼ dilution of cDNA, 0.4 µM of each primer and 12 µL qPCR Mastermix. Conditions for amplification were: 2 min at 50 °C, 10 min at 95 °C, followed by 40 cycles of 15 s at 95 °C, 1 min at 58 °C, and dissociation curve analysis of 15 s at 95 °C, 1 min at 58 °C, 15 s at 95 °C. Each biological sample was analyzed for eight primer sets and for a housekeeping gene (Actin), using three biological samples per treatment and three technical replicates per biological sample. Data were analyzed using Design & Analysis 2.6.0 software provided with the instrument QuantStudio 7 Pro (Thermo Fisher Scientific, Waltham, MA). The list of primers used for qPCR is shown in Supplementary Table 5.

### Melanin quantification

To determine melanin production, RED, TAN, BROWN and NRRL 64463 CP isolates were grown on PDA medium incubated at 26 °C ± 0.5 °C in the dark for 17 days. Biomass of two plates per culture of each isolate were placed into separate 50 mL centrifuge tubes and stored at -80 °C until processing. The samples were lyophilized at 3 Pa for 16 h, biomass weighted with a 5-decimal place accuracy in a XPR105DR, XPR Analytical balance (Cat. # 30355342, Mettler Toledo, Columbus, OH), and processed for the removal of AQ that interfere with the quantification of melanin as follows: the tubes were loaded with 16 zirconium beads (12 of 2.8 mm dia. and 4 of 6.5 mm dia.) and 15 mL of ethyl acetate, sealed with matching caps and the fungal mass was pulverized in an OMNI Bead Ruptor (OMNI International, cat. # 19-042E, Kennesaw, GA) at 5.5 m/sec for 45 s. The slurry obtained was centrifuged at 1,800 × *g* for 8 min in a Model 6755 LSE compact centrifuge (Corning Life Sciences); the supernatant was carefully removed with a glass pipette. Extraction of AQ was repeated three more times with 15 mL ethyl acetate followed by centrifugation and removal of the supernatant. The precipitate was allowed to dry in a fume hood at ambient temperature for 3 h. After removal of AQ, melanin was extracted according to reported protocols^27,28^, with slight modifications as follows: the finely ground biomass (100 mg/sample) was placed in 25 mL Erlenmeyer flasks, 20 mL of freshly prepared 0.5 M NaOH were added, each flask was sealed with three layers of parafilm, and incubated overnight in a shaker incubator at 25 °C and 150 rpm. The extract was then centrifuged at 7,200 *g* and the supernatant was vacuum filtered using a glass fiber filter type E (Gelman Instrument Company, Ann Arbor, MI) as prefilter and a 0.22 μm PVDF membrane. The filtrate was placed in 50 mL polypropylene centrifuge tubes with a small stirrer, added 1 N HCl until reaching pH: 1.5, and allowed to rest for one hour at ambient temperature. Then, the tubes were centrifuged at 7,200 *g* and 15 °C for 70 min to precipitate the melanin. The pellet was washed once with 3 mL 0.1 N HCl, centrifuged again at 7,200 *g* for 15 min, then resuspended first in 300 µL 0.1 N HCl that were transferred to a pre-weighed 4 mL glass vial, another 200 µL of 0.1 N HCl were used to collect the remainder and added to the glass vial. Vials containing 500 µL of melanin suspension were frozen at -80 °C for 10 min and lyophilized. The final weight of the precipitate was measured in a 5-decimal place balance XPR105DR. Results were expressed as mg of melanin per g of dry weight of fungal biomass. Statistical analysis of the results was performed as mean comparison by Tukey’s test, using SYSTAT (Sigma Plot v. 15).

### Presence of chitin, or 1–3 and 1-4-β-glucans

Standard β-glucan (CAS 9012-72-0, Prod. No. 346210) was purchased from Millipore (Sigma-Aldrich, St. Louis, MO). Two mg of β-glucan were dissolved in 100 µL sterile distilled water. Twenty µL of that solution were placed in 380 µL sterile distilled water, added 2 µL of 5 mM calcofluor white (Cat. # 29067, Biotium Inc., Fremont, CA), wrapped in aluminum foil and incubated for 20 min at ambient temperature ~ 22 °C. Four technical replicates of 50 µL of the calcofluor-treated solution were applied to a 96-well black ultra-thin flat clear bottom plate (Corning; Cat. # 3615, Corning, NY) containing 50 µL per well of sterile distilled water and proceeded to make two-fold dilutions of the sample. The plate was read in a Synergy HTX multi-mode reader, BioTek (Agilent, Santa Clara, CA), using a tungsten light excitation filter 360/40 nm, emission filter 460/40 nm and 10 reads per data point, using Gen 5 Software 2.09.1. Reads of the first 6 dilutions followed a linear relationship and the fluorescence values were used as reference for calculations. The four CP isolates were grown for 12 days on PVDF membranes at 25 °C in the dark, then the biomass was harvested, placed into 50 mL centrifuge tubes at -80 °C overnight before lyophilizing. From each culture, three biological replicates of 20 mg dry biomass were placed into 2 mL OMNI tubes containing 3 × 2.8 mm zirconium beads. Sterile water, 400 µL, was added to each tube and the biomass was homogenized in an OMNI Bead Ruptor at 5 m/s for 30 s three times with 1 min rest between cycles. Two µL of 5 mM calcofluor white were added to each tube, mixed, covered with aluminum foil, and incubated at room temperature ~ 22 °C for 20 min. After incubation, the tubes were centrifuged for 5 min at 20,800 *g* to precipitate fungal biomass, then 380 µL of supernatant were removed to reduce background fluorescence and replaced with 380 µL sterile distilled water. A total of 50 µL of sterile distilled water were added to the wells of a 96-well black ultra-thin flat clear bottom plate. Three technical replicates per biological sample were prepared by adding 50 µL of culture homogenate treated with calcofluor, and consecutive two-fold dilutions were prepared on the 12 well-columns of each plate. A tube without fungal biomass was processed with calcofluor in the same manner and used as control. Reads from the control wells at each dilution were subtracted from the reads of each sample at the corresponding dilutions to account for fluorescence background. Slopes and intercepts of the Log_2_ transformed fluorescence curves for the first six dilutions, as well as maximum fluorescence on the first dilution were subjected to analysis of variance (ANOVA) and mean comparisons by Tukey’s test using SYSTAT (Sigma Plot V. 15).

### Pathogenicity

Twelve-day old cultures of the three color-CP isolates and NRRL 64463 were grown on PDAP medium, that is PDA medium supplemented with 25 mL/L of peanut-leaf extract (PDAP). The leaf extract was prepared by blending 300 g Georgia 06G leaves in 500 mL distilled water, filter sterilized through 0.22 μm and stored at -20 °C until use. The leaf extract was added after cooling the autoclaved PDA medium to 55 °C, thoroughly mixed and then poured into Petri dishes. Six leaves, second and third from the top of grow-light illuminated indoor-grown peanut cultivars Georgia-06G and AU-NPL17, were used in detached-leaf pathogenicity assays^58^. A total of 24 inoculation sites were used such that four leaflets of six leaves per cultivar received 3 µL of inoculum. Observations were performed every other day, starting 5 days after inoculation, though symptoms were confirmed after 2–3 weeks, and the experiment was repeated.

### Chemical analysis

#### Reagents and materials

HPLC-grade organic solvents used for fungal mass extraction and for preparation of the mobile phase were purchased from Fisher (Suwanee, GA). HPLC-grade water was prepared in the laboratory with a ZD20 four-bowl Milli-Q water system (Millipore). Formic acid (LS/MS, Optima) was purchased from Fisher Scientific (Cat. # A117-50). Aliquots of anthraquinones, nidurufin, averantin, averufin, averufanin, and aversin were a generous gift from Dr. Kimiko Yabe (Fukui University of Technology, Japan). Versicolorin B, versicolorin A, norsolorinic acid, versiconol, versiconol acetae, O-methylsterigmatocystin, sterigmatocystin, dihydro sterigmatocystin, dihydro O-methylsterigmatocystin, dihydroxyaflavinine, asparason A, aflatrem, emodin, aflatoxins B_1_, B_2_, G_1_, G_2_, M_1_, and B_2a_ were made available as a part of the fungal secondary metabolites collection at the NPRL. The chromatographic purity of any of the listed above compounds exceeded 92%. Ergosterol (CAS_No. 57-87-4, Prod No. PHR1512) and squalene (CAS No. 111-02-4, Prod No. S3626, ~ 98%) were purchased from Millipore (Sigma-Aldrich Inc., St. Louis, MO).

#### Sample preparation

Five biological replicates for each of the three colored CP isolates, RED, TAN, BROWN and NRRL 64463 grown at 25 °C for 12 days with 16/8 h photoperiod, and another five replicates grown in darkness, were used for chemical profiling of secondary metabolites/pigments. After incubation, the fungal biomass was removed from each of the disks with a spatula and separately placed into 50-mL polypropylene centrifugal tubes (Corning 430290, Millipore-Sigma CLS430290) followed by lyophilization at 3 kPa for 16 h. Then, the tubes were charged with 16 zirconium ceramic beads (12 of 2.8 mm diameter and 4 of 6.5 mm diameter) and 25 mL mixture of methanol - ethyl acetate (1:2, v/v). Tubes were securely sealed with matching caps and the fungal mass was pulverized in an OMNI Bead Ruptor at 5.5 m/sec for 45 s. The slurry was centrifuged at 1,800 × *g* for 3 min in a Model 6755 LSE Compact Centrifuge (Corning Life Sciences); the supernatants were collected with glass pipettes into 250 mL round bottom flasks. Extraction/pulverization of the precipitates was repeated two more times with 20 mL ea. of the methanol - ethyl acetate mixture (1:2, v/v) followed by centrifugation and collection of the supernatants. The 3 extracts from the same samples were combined and evaporated with a Rotavapor-R rotary evaporator (Brinkmann Instruments, Westbury, NY) at 40 °C to dryness followed by addition of 10 mL of precisely measured methanol to each flask with dry residues. Then the flasks were capped with matching stoppers and sonicated in a Model T-9 ultrasonic bath (L&R, Kearny, NJ) for 30 s; the solution was transferred to 15 mL glass vials (Thermo Scientific, cat. # B7800-4) with matching caps and centrifuged in the same centrifuge under the same as above conditions for 3 min. About 0.25 mL aliquots of the centrifuged samples were filtered into 400 µL polypropylene autosampler vials (Fisher Scientific, cat. # C4010-11) through 10 × 10 mm pieces of glass fiber filter paper firmly placed into the narrow part of Pasteur pipettes; nitrogen gas from a compressed nitrogen tank was used to expedite filtration. Vials were sealed with matching caps with PTFE septa (Fisher Scientific, cat. # C4010-60 A). Between 1/10th of a µL and 3 µL of filtered extracts were injected into the HPLC system.

#### HPLC-DAD-MS analysis

Separations of authentic standards and the fungal extracts were performed using a tandem HPLC − MS system equipped with Vanquish Split Sampler FT (part # VF-A10-A), Diode Array Detector FG (part # VF-D11-A) covering the 210–800 nm range, Quaternary Pump F (part # VF-P20-A) (all Thermo Scientific, San Jose, CA), and a 100 mm × 4.6 mm i.d., 3.5 μm XSelect HSS C18 analytical column (Waters). H_2_O (A), MeOH (B), and 1% HCOOH in H_2_O (C) were used in the following gradient: initial conditions, 31% A/65% B/4% C, changed linearly to 0% A/96% B/4% C in 4 min, held isocratic for 10 min, and then changed to initial conditions in 0.01 min and held for 3 min before the next injection. The flow rate was 0.5 mL/min. The column was maintained at 40 °C. MS analysis was performed using an LTQ XL ion trap mass spectrometer equipped with an APCI interface and operated with Xcalibur version 4.4.16.14 software (Thermo Scientific, San Jose, CA). The data were acquired in the full-scan mode (MS) from m/z 100 to 2000. The capillary temperature was 130 °C, APCI vaporizer temperature 230 °C, sheath gas flow 50 units, auxiliary gas flow 5 units, capillary voltage 9 V, and source voltage 6.0 kV. In MS^2^ analyses, the [M + H] ^+^ ions observed for each chromatographic peak in full-scan mode, were isolated and subjected to source collision induced dissociation (CID) using He gas. In all CID analysis, the isolation width, relative fragmentation energy, relative activation Q, and activation time were 1.0, 20 or 35%, 0.25, and 30 ms, respectively. Concentrations of all identified compounds in the extracts were calculated by reference to peak areas (calibration curves) of corresponding pure standards at their UV absorption maxima.

#### Statistical analysis

Results expressed in µg/g from five biological replicates for each compound identified were analyzed by analysis of variance (ANOVA), and means were compared by Tukey’s test using SYSTAT (Sigma Plot V. 15).

### Mating type

Previously we reported that annotated Contig_298^1^ contains the MAT 1 locus^16^. Thus, we explored the possibility that color-CP isolates could have different mating types. For this, we first performed de novo assemblies of their genomes, and BLAST^78^ of Contig_298 to those assemblies. Then, using Whole Genome Alignment (WGA)^79,80^ with minimum initial seed 40 bp, and 100 bp minimum alignment block length, compared the BLAST results with the genomes of IPAVE0302^16^ and CBS 151044^23^. Further iterations of BLAST analysis of the MAT locus using *Cladosporium fulvum* Race 5 (Syn. *Fulvia fulva*) genome as a model^22^ in combination with WGA were performed to compare all six isolates and characterize the MAT1-1 and MAT1-2 MAT + sexual cell fertilization-promoting factors. All the analyses were performed using CLC Genomics Workbench 23.0.2.

### Resistance to xenobiotics

The effect of xenobiotics on growth area and density of CP isolates was evaluated. The four xenobiotics tested were: caffeine (CAF)(CAS Number: 58-08-2) Sigma-Aldrich, tebuconazole (TEB)(CAS Number: 107534-96-3), prothioconazole (PROT)(CAS Number: 178928-70-6), and chlorothalonil (CHLOR)(2,4,5,6-Tetrachloroisophthalonitrile; CAS Number: 1897-45-6), was tested at four different concentrations against the three CP color isolates and the reference NRRL 64463. Given its low solubility, CAF was added to hot PDA medium immediately after autoclaving and thoroughly mixed before pouring plates. The final concentrations used for CAF were 2, 4, 6 and 10 mM. TEB, PROT, and CHLOR were added after cooling to 55 °C the autoclaved PDA medium and thoroughly mixed before pouring plates. The final concentrations used for TEB, PROT and CHLOR were 0.3, 1.0, 3.3 and 10.0 mg a.i./L. The CP isolates grown on PDA medium were used as controls. For testing the response to xenobiotics, the CP isolates were placed on top of Polyvinylidene fluoride (PVDF) membranes 0.45 μm (HVLP04700, Merck Millipore, Cork, Ireland). The PVDF membranes were autoclaved for 10 min before use, then single membranes were placed on the surface of the medium of each plate. One cm^2^ of 12-day old culture grown on PDA was placed in sterile test tubes containing 2 mL sterile distilled water then ground with a LabGEN 125 Tissue Homogenizer. For each concentration of xenobiotic and fungal isolate, three plates were prepared placing four undisturbed 20-µL-drops of homogenate on each PVDF membrane. The plates were incubated in darkness at 26 °C for 5 days, then three randomly selected drops were photographed using backlight in a stereoscope Nikon SMZ800 and Leica Application Suite software (LAS V4.3). Areas and density of growth for each drop were estimated using ImageJ software (https://imagej.net/ij/, National Institutes of Health, USA) as described before^9^. The experiments were repeated once, data were analyzed by ANOVA using SYSTAT V.15 and means compared by Tukey’s test.

## Electronic supplementary material

Below is the link to the electronic supplementary material.

Supplementary Material 1

## Data Availability

The datasets generated and/or analyzed during the current study are available in NCBI GenBank, genomes SRA: SRR24707293, SRR24718870, SRR24718913; transcriptomes SRA: SRR25052186, SRR25057223, SRR25054762, SRR25144991, SRR25182278, SRR25231499, SRR25234020, SRR25237168. Further details listed in Supplementary Tables 1 and 3.
