# Supplementary material for: Characterizing phenotype variants of Cercosporidium personatum, causal agent of peanut late leaf spot disease, their morphology, genetics and metabolites
Source: Sci Rep. 2025 Jan 9;15:1405. doi: 10.1038/s41598-025-85953-9 (PMC11718120; doi:10.1038/s41598-025-85953-9)

Supplementary Table 1. Summary genomic DNA sequencing and assembly of three isolate morphotypes of *Cercosporidium personatum*. NCBI Bioproject: PRJNA975561. SRA: Sequence Read Archive; Q: quality score $Q = -10 \times \log_{10}(e)$ where e is the probability of wrong base calling; N50: 50% of all nucleotides lie in contigs at least this length in base pairs (bp).

Sample names	**NPR22, RA1, RED**	**NPT22, RA2, TAN**	**NPO22, RA3, BROWN**
Biosample	SAMN35332042	SAMN35332281	SAMN35332338
SRA	SRR24707293	SRR24718870	SRR24718913
Raw Reads	41,254,146	21,807,088	24,338,694
Raw bases	6.19Gb	3.27Gb	3.65Gb
Q20 (%)	96.98	93.37	92.17
Q30 (%)	92.55	88.59	87.18
Clean sequences (>100bp)	35,896,956	16,254,168	17,849,592
Genome size (bp)	31,386,073	31,050,225	31,325,865
Number of contigs	1418	1884	1716
N75 (bp)	30,136	20,834	23,414
N50 (bp)	63,509	41,117	47,736
N25 (bp)	136,119	76,267	93,494
Minimum length (bp)	1006	1000	1000
Maximum length (bp)	436,830	238,699	248,411
Average length (bp)	22,134	16,481	18,255
GC %	52.40	52.65	52.54
Reads not mapped	3,250,500	1,918,011	2,109,651

Supplementary Table 2. Summary of genome-wide variants detected on three color isolates of *Cercosporidium personatum* (present work) and the reference genome NRRL 64463 (Bioproject: PRJNA975561). SNP: single nucleotide polymorphisms; MNP: multi-nucleotide polymorphisms; InDel: Insertion/Deletion variants; *: amino acid (AA) changes not observed in the reference genome NRRL 64463.

	Total	SNP	MNP	InDel	Ratio SNV/kb	Ratio InDel/kb	Variants resulting in AA change	AA changes unique to each culture
RED	40467	35332	3074	2061	1.39	0.07	2990*	812
TAN	40359	35231	3096	2032	1.39	0.07	3103*	925
BROWN	48628	42991	3239	2398	1.68	0.09	3960*	1781
NRRL 64463	18465	16647	1279	539	0.65	0.02	612	549

Supplementary Table 3: RNA sequencing of four *Cercosporidium personatum* isolates. Average GC in all libraries: 54.5 %. *: from Arias *et al*. 2023. Data Linked to Bioproject PRJNA988430.

ACCESSION	Library ID	File type FASTA.GZ	Sample name	Reads	Bases (nt)
SRR25052186*	CPDP-13A_1	CP_1_S1_L001_R1_001	CPDP-13A	100,794,012	15,219,895,812
		CP_1_S1_L001_R2_001			
SRR25057223*	CPDP-13A_2	CP01_2_S2_L001_R1_001	CPDP-13A	116,662,050	17,615,969,550
		CP01_2_S2_L001_R2_001			
SRR25054762	NPR22_1	NPR22_1_S3_L001_R1_001	NPR22 (RED)	127,705,926	19,283,594,826
		NPR22_1_S3_L001_R2_001			
SRR25144991	NPR22_2	NPR22_2_S4_L001_R1_001	NPR22 (RED)	116,593,220	17,605,576,220
		NPR22_2_S4_L001_R2_001			
SRR25182278	NPT22_1	NPT22_1_S5_L001_R1_001	NPT22 (TAN)	131,705,044	19,887,461,644
		NPT22_1_S5_L001_R2_001			
SRR25231499	NPT22_2	NPT22_2_S6_L001_R1_001	NPT22 (TAN)	143,788,770	21,712,104,270
		NPT22_2_S6_L001_R2_001			
SRR25234020	NPO22_1	NPO22_1_S7_L001_R1_001	NPO22 (BROWN)	149,279,110	22,541,145,610
		NPO22_1_S7_L001_R2_001			
SRR25237168	NPO22_2	NPO22_2_S8_L001_R1_001	NPO22 (BROWN)	145,696,964	22,000,241,564
		NPO22_2_S8_L001_R2_001			

Supplementary Table 4. List of orthologous genes of *Cercosporidium personatum* involved in Dothistromin/Aflatoxin/Anthraquinone biosynthesis. Annotations correspond to the transcripts in Arias et al. 2023.

**Transcript annotated in published genome (Arias et al 2023)**	**Chromosome**	**Gene**	**Description**
augustus_masked-cont_469-0.5	12	*hexA*	*hexA*, *aflA*, Fatty acid synthase, fas-2, Dothistromin pathway
augustus_masked-cont_469--0.2	12	*hexB*	Fatty acid synthase beta subunit hexB
augustus_masked-cont_469-0.1	12	*hexB*	hexB, fas-1, fatty acid synthase beta subunit
augustus_masked-cont_291-0.10	12	*pksA*	*pksA*, *aflC*, polyketide synthase, Dothistromin pathway, conidial yellow pigment
augustus_masked-cont_469-0.4	12	*hypC*	Anthrone oxidase, from polyketide to Norsolorinic Acid (possible DS12)
augustus_masked-cont_242-0.9	12	*nor1*	norsolorinic acid ketoreductase nor1 in *Fulvia fulva*
augustus_masked-cont_186-0.4	10	*AvnA*	Averantin hydroxylase [*Fulvia fulva*]; Cytochrome P450 monooxygenase orf2 [*Fulvia fulva*]

augustus_masked-cont_746-0.7	4	*AvnA*	Averantin hydroxylase [*Fulvia fulva*]_Cytochrome P450 monooxygenase lcsI _AVN to HAVN then to AVF by is also Pisatin Demethylase
augustus_masked-cont_85-0.29	3	*adhA*	5'-hydroxyaverantin dehydrogenase [*Fulvia*] HAVN dehydrogenase_catalizes the conversion of HAVN to Aferufin
augustus_masked-cont_242-0.6	12	*adhA*	5'-hydroxyaverantin dehydrogenase, short-chain dehydrogenase/reductase prx4 (*Fulvia*), (AVN>HAVN>adhA to AVF)
augustus_masked-cont_291-0.15	12	*cypA*	*cypA*, *aflV*, CytP450 monooxygenase, Averufin monooxygenase (cypX), AVR->HVN, Dothistr pathway
snap_masked-cont_291-0.25	12	*avfA*	*avfA*, *aflI*, oxidase Dothistr pathway, converts Averufin (AVR) to versiconal acetate (VHA)
augustus_masked-cont_677-0.42	7	*avfA*	Averufin oxidase A [*Fulvia fulva*]
augustus_masked-cont_1051-0.7	12	*moxA*	*moxA*, *aflW*, Dothistr pathway, Hydroxyversicolorone monooxygenase
augustus_masked-cont_472-0.8	12	aflJ	aflJ or estA, Dothistromin biosynthesis regulatory protein aflJ [*Fulvia fulva*] Esterase? VHA =>VAL before VerB
augustus_masked-cont_469-0.3	12	*vbsA*	*vbsA*, *aflK*, versicolorin B synthase
augustus_masked-cont_242-0.7	12	*verB*	*verB*, *aflL*, versicolorin B desaturase, oxidative desaturation of VERB to VERA
augustus_masked-cont_784-0.46	12	*dotA*	dotA, *aflM*, *ver-1*, Versicolorin reductase, ketoreductase, versicolorin A (VERA) reductase, necessary to converts VERA (with aflN and aflY) to DMST (Demethylsterigmatocystin)
augustus_masked-cont_51-0.47	10	*dotA*	dotA, *aflM*, *ver-1*, Versicolorin reductase, ketoreductase, versicolorin A (VERA) reductase, necessary to converts VERA (with aflN and aflY) to 5,8 deoxydothistromin or DMST (Demethylsterigmatocystin)
snap_masked-cont_784-0.61	12	*dotB*	dotB, Dothistromin biosynthesis peroxidase
augustus_masked-cont_784-0.47	12	*dotC*	dotC, *aflT* toxin pump, MFS transporter, Dothistr pathway
augustus_masked-cont_784-0.33	12	*dotD*	dotD, *aflC* thioesterase, Dothistr pathway
snap_masked-cont_65-0.6	12	*ordB*	Deoxydothistromin to dothistromin
augustus_masked-cont_472-0.0	12	*aflR*	aflR: Dothistromin biosynthesis regulatory protein aflR [*Fulvia fulva*]
augustus_masked-cont_759-0.5	12	*DS30*	DS30 within Dothistr pathway
augustus_masked-cont_759-0.4	12	*DS31*	DS31, translation elongation factor, within Dothistr pathway
augustus_masked-cont_678-0.11	12	*DS33*	DS33, within Dothistr pathway
augustus_masked-cont_291-0.18	12	*MFS*	putative MFS-type transporter, close to GlyHydro, not annotated in literature
augustus_masked-cont_1051-0.6	12	*DS25*	DS25 within Dothistr pathway, amino acid permease
augustus_masked-cont_169-0.17	12	*DS16*	DS16 within Dothistr pathway

augustus_masked-cont_1053-0.29	1	*AFAR*	*AFAR*, aflatoxin-aldehyde reductase, converts AFB1-dihydrodiol to AFB1-dialcohol (detoxification)

Supplementary Table 5. Primers used for quantitative real-time PCR (qRT-PCR) to analyze gene expression in three color variants of *Cercosporodium personatum* and the reference NRRL 64463. *: gene annotation from Arias *et al*. 2023: am = augustus_masked; sm: snap_masked; ctg = contig; -- = processed-gene.

**Stock**	**Gene**	**Gene model***	**Primer ID**	**Forward 5' → 3'**	**Reverse 5' → 3'**	**Position**	**Length (bp)**
668/669	actin	am-ctg_582--0.4	ACT_int2	CACGGTGTTGTCACCAACTG	GTGCGTTGAAGGTCTCGAAG	787-958	172
672/673	*pksA*	am-ctg_291--0.10	291_pksA_int1	AAGGACAACCCGATCCTGAC	GAAGGCAGGGCTCAACTTTC	88-249	161
676/677	*vbsA*	am-ctg_469--0.3	469_vbs_int1	ACTGAAGTGCCCATGTACCTC	GTCTTGCCCTGCATGTAGAAC	358-494	136
678/679	*dotA*	am-ctg_784--0.46	784_dotA_int1	TCAGGTTGCGAAGATGATGG	GTTGGAGGACGTGAGGATAATG	219-438	219
682/683	*verB*	am-ctg_242--0.7	242_verB_int1	GAGACACTGCGCATGTATCC	TGTCGATGTGGCGTTCTG	1144-1330	186
686/687	*avfA*	sm-ctg_291--0.25	291_avf	CCGGACCTCGAACTCAACATC	GCACATGAAGGCGACATGG	79-246	167
688/689	*pks1*	sm-ctg_715--0.94	715_pks1_1	GTCGTCTTGCGCTAGTTACTGC	CGTTCTCAGCGGCGTTTATCTC	1373-1519	146
690/691	*pks1*	sm-ctg_715--0.94	715_pks1_2	TGCTGCTTTGTGCCCATC	CTTGTGCGGGTTGTGGAATC	3981-4141	160
692/693	*t4hnr*	am-ctg_51--0.47	51_t4hnr_int1	TCCAGGTGGTATCAAGAC	CAGACAACACGAGCAATG	690-845	155

Supplementary Figure 1

Growth curves of four CP isolates

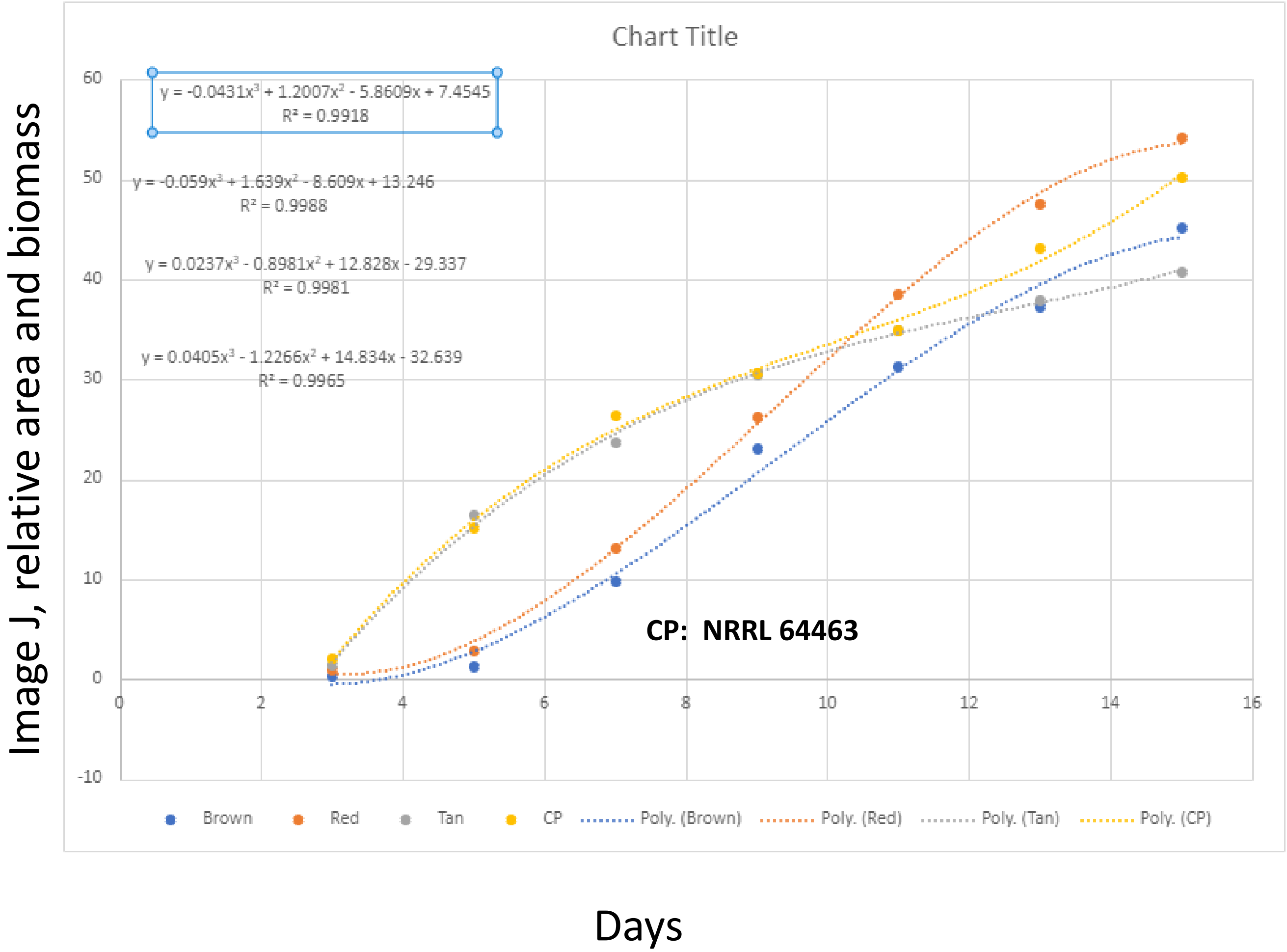

Supplementary Figure 2

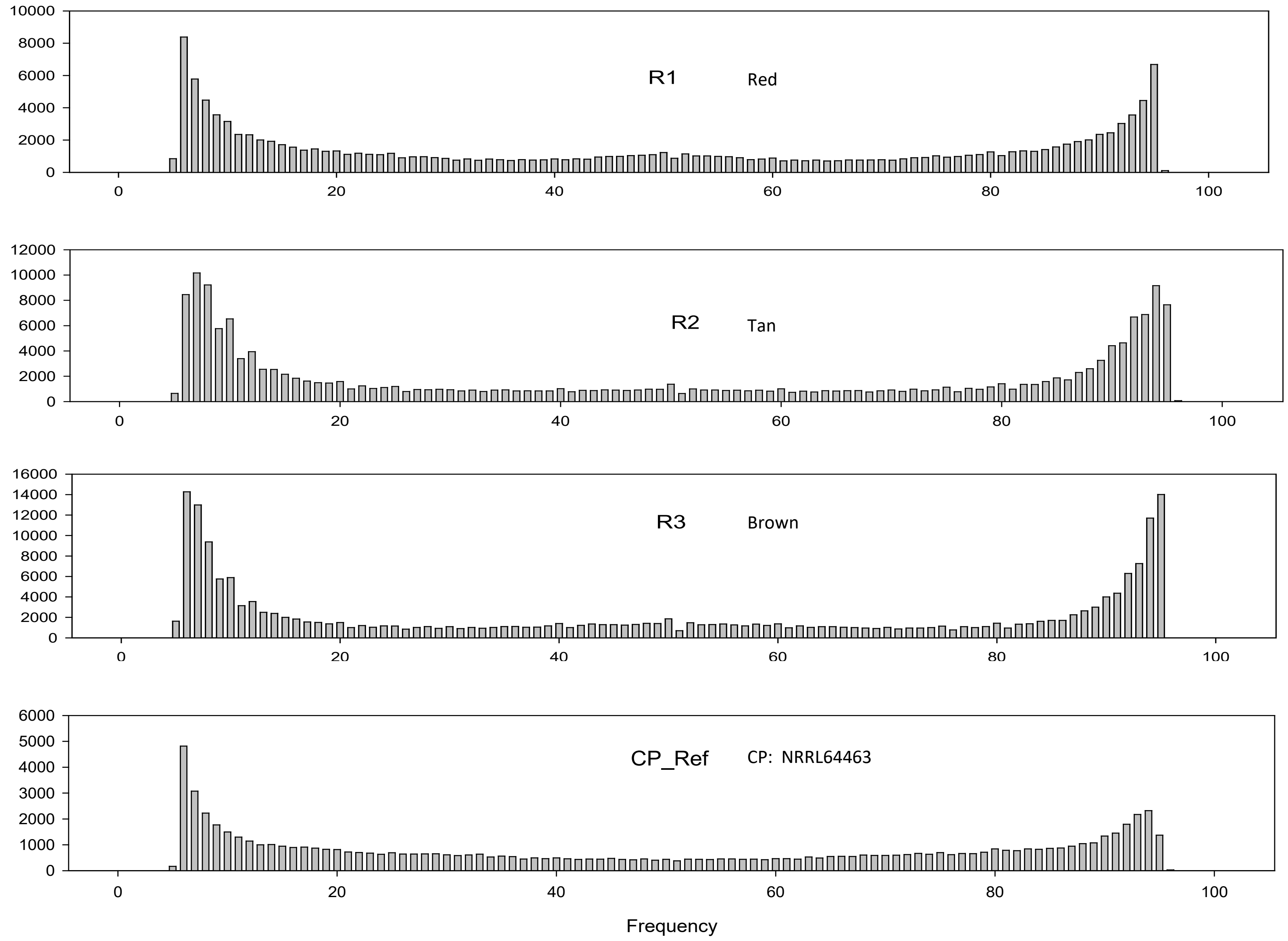

Supplementary Figure 3

dbCAN

Differential expression dbCAN2 genes		Due to TYPE					NRRL64463		Brown		Red		Tan	
Name	Max grou	Fold chan	Log fold c	P-value	FDR p-valu	Bonferron	CP01	CP01-2	NPO22	NPO22-2	NPR22	NPR22-2	NPT22	NPT22-2
augustus_masked-contig_742-processed-gene-0.15-mRNA-1	4228.24	-264.39	-8.05	0	0	0	-3.31535	-3.34987811	1.92140582	1.92273674	-5.70436644	-6.41113563	-3.43526604	-3.34004237
augustus_masked-contig_19-processed-gene-0.1-mRNA-1	17.32	18.42	4.2	0	0	0	-2.83094	-2.92018742	1.64354422	1.6681108	-2.20945337	-2.44625623	-0.94641896	-0.98158796
augustus_masked-contig_552-processed-gene-0.8-mRNA-1	842.02	15.87	3.99	0	0	0	-3.1343	-2.577783	1.56895604	1.40033837	-1.23454592	-1.04747928	-1.01673004	-0.4960321
maker-contig_379-augustus-gene-0.67-mRNA-1	65.21	-10.75	-3.43	0	0	0	-1.26442	-0.93165599	1.50543406	1.54510471	-1.6562996	-2.03245663	-1.2264065	-1.67552209
augustus_masked-contig_469-processed-gene-0.3-mRNA-1	9452.35	-20.45	-4.35	0	0	0	-2.63421	-2.28041477	1.42782748	1.41824355	-2.85562675	-2.8824369	0.01486558	-0.02041429
augustus_masked-contig_346-processed-gene-0.5-mRNA-1	75.31	11.85	3.57	0	0	0	-2.9102	-2.5917633	1.40257533	0.91945952	-1.28705764	-0.4434833	-0.29786439	0.27184823
augustus_masked-contig_72-processed-gene-0.13-mRNA-1	75.39	-12.31	-3.62	0	0	0	-2.00343	-1.83715988	1.38515265	1.44469077	-1.79227859	-2.5244841	-0.23466657	-0.27879159
augustus_masked-contig_563-processed-gene-1.99-mRNA-1	76.03	-10.85	-3.44	0	0	0	-1.3649	-0.89517083	1.36365522	1.36413823	-0.42265643	-0.91101511	-1.7743546	-1.48891422
augustus_masked-contig_499-processed-gene-0.95-mRNA-1	15.45	-9.65	-3.27	0	0	0	-0.78269	-0.80128041	1.33466408	1.34007544	-2.2059043	-1.64081574	-0.55681146	-0.80128041
augustus_masked-contig_187-processed-gene-0.16-mRNA-1	787.18	-37.06	-5.21	0	0	0	-0.32537	-0.43501166	1.25566265	1.25829298	-3.54371421	-4.19784244	-0.45722237	-0.29743812
augustus_masked-contig_327-processed-gene-0.61-mRNA-1	211.51	8.74	3.13	0	0	0	-2.1931	-2.08938665	1.21778284	1.43346369	-0.59671725	-1.1613791	-0.33247723	-0.69667968
augustus_masked-contig_272-processed-gene-0.23-mRNA-1	983.88	-14.85	-3.89	0	0	0	-2.61571	-2.3493537	1.16530765	1.13376217	-2.68031826	-2.68364337	0.52409398	0.53984491
augustus_masked-contig_344-processed-gene-0.1-mRNA-1	160.67	-8.04	-3.01	0	0	0	-1.38497	-1.09212422	1.08736381	1.23322369	-1.55473917	-2.00701301	0.20396368	-0.10279836
snap_masked-contig_238-processed-gene-0.31-mRNA-1	929.11	-10.27	-3.36	0	0	0	-1.14991	-1.16224421	1.05801105	0.75154217	-2.36566543	-2.44289085	0.34852578	0.75332791
augustus_masked-contig_416-processed-gene-0.14-mRNA-1	378.77	-20.52	-4.36	0	0	0	-0.04935	0.09475968	0.58380685	0.40590334	-3.62401528	-3.97845985	0.53975272	0.62644296
augustus_masked-contig_16-processed-gene-0.30-mRNA-1	60.99	404.41	8.66	0	0	0	-7.33204	-7.95564991	-2.55067683	-2.74619654	-2.11286803	-2.5864161	1.84475993	1.8798742
augustus_masked-contig_89-processed-gene-0.9-mRNA-1	262.12	16.33	4.03	0	0	0	-1.88002	-1.75267783	-0.86166024	-0.62851378	-2.61698209	-3.06421075	1.59215424	1.55426887
augustus_masked-contig_254-processed-gene-0.68-mRNA-1	267.61	23.11	4.53	0	0	0	-2.6875	-2.48242954	-0.30548025	-0.28482608	-3.32493058	-3.42423466	1.55375513	1.53956204
augustus_masked-contig_403-processed-gene-0.15-mRNA-1	18.85	10	3.32	0	0	0	-2.79008	-2.63941028	-0.41156531	0.12503605	-1.69696753	-1.73498794	1.48294156	1.29193335
augustus_masked-contig_307-processed-gene-0.9-mRNA-1	84.64	24.97	4.64	0	0	0	-1.2508	-1.01954924	-0.25315043	0.03005552	-3.57742883	-3.78185768	1.40977338	1.27081354
snap_masked-contig_213-processed-gene-0.66-mRNA-1	4979.99	16.95	4.08	0	0	0	-3.23115	-3.21975628	0.21057126	0.40305211	-3.05025097	-3.19916554	1.38716286	1.29952428
augustus_masked-contig_356-processed-gene-0.16-mRNA-1	127.43	14.99	3.91	0	0	0	-0.34714	-0.66451957	-2.54532065	-3.1136892	-1.21873118	-2.10637205	1.38564629	1.5951901
augustus_masked-contig_522-processed-gene-0.11-mRNA-1	71.33	8.63	3.11	0	0	0	-0.18203	0.19403129	-0.59754605	-0.79630238	-2.22678419	-2.51491993	1.16609166	1.07400058
snap_masked-contig_738-processed-gene-0.2-mRNA-1	105.36	12.73	3.67	0	0	0	-0.0569	-0.40091445	-2.60037276	-3.22613211	-0.18069488	-0.68229097	0.99385563	1.32917042
augustus_masked-contig_456-processed-gene-0.9-mRNA-1	81.35	-10.91	-3.45	0	0	0	0.809077	0.96688504	-1.61435507	-1.45349154	-2.05275656	-2.22065737	0.74869776	0.55159375
augustus_masked-contig_118-processed-gene-0.49-mRNA-1	559.88	-14.89	-3.9	0	0	0	1.113758	1.36882878	-1.14850338	-1.19473284	-1.9396315	-2.5562979	0.16881855	-0.30309236
augustus_masked-contig_390-processed-gene-0.3-mRNA-1	519.56	-11.9	-3.57	0	0	0	1.14919	1.29488877	-1.292341	-1.26151093	-1.79214472	-2.06458227	0.11779597	-0.16770657
snap_masked-contig_219-processed-gene-0.130-mRNA-1	80.71	-16.99	-4.09	0	0	0	1.029002	1.1906547	-2.50799531	-2.72489276	0.48972014	0.43497857	-1.39814813	-2.22239242
augustus_masked-contig_1049-processed-gene-0.6-mRNA-1	57.82	8.59	3.1	0	0	0	-2.83476	-2.10465718	-1.37371375	-1.64439042	1.25792825	0.85417565	0.11425243	0.71359593
snap_masked-contig_141-processed-gene-0.6-mRNA-1	262.41	9.39	3.23	0	0	0	-2.84122	-2.20533169	-2.13487201	-2.22145135	1.31784563	0.86505764	0.18882337	0.8051498
augustus_masked-contig_431-processed-gene-0.32-mRNA-1	33.95	9.21	3.2	0	0	0	-2.63262	-1.94901607	-0.4179339	-0.47508488	1.36940181	1.33579885	-0.98102824	-1.00743276
augustus_masked-contig_22-processed-gene-0.52-mRNA-1	16.21	-11.03	-3.46	0	0	0	-2.19515	-2.21174518	-0.07439543	-0.44122702	1.38216134	1.39891783	-2.1500597	-1.28425768
augustus_masked-contig_89-processed-gene-0.20-mRNA-1	45.96	-10.81	-3.43	0	0	0	-1.99854	-1.77389962	-0.20462609	-0.042318	1.38438324	1.26434334	-1.88161773	-1.58073791
snap_masked-contig_186-processed-gene-0.125-mRNA-1	10180.76	-9.78	-3.29	0	0	0	-0.71776	-0.39098311	-1.74596652	-2.08860733	1.47438235	1.37691858	-1.66575679	-1.30346458
snap_masked-contig_188-processed-gene-0.49-mRNA-1	107.99	-10.02	-3.32	0	0	0	-2.04392	-1.57443074	-0.84880965	-1.28230995	1.49939031	1.53299834	-1.41495784	-1.47705532
augustus_masked-contig_538-processed-gene-0.14-mRNA-1	200.89	-14.4	-3.85	0	0	0	-2.26091	-2.36647296	-0.62700142	-0.77943911	1.54109947	1.54868525	-2.0974125	-1.74669569
augustus_masked-contig_547-processed-gene-0.59-mRNA-1	150.07	-8.48	-3.08	0	0	0	-2.19203	-1.59051551	-1.44535444	-1.3308717	1.56216324	1.51037417	-1.11718669	-1.24612136
augustus_masked-contig_45-processed-gene-0.26-mRNA-1	139.8	12.73	3.67	0	0	0	-3.04369	-2.1142779	-1.12444102	-1.25773069	1.56340282	1.43967922	-1.10610142	-0.64784681
snap_masked-contig_633-processed-gene-0.7-mRNA-1	180.2	-9.39	-3.23	0	0	0	-2.06033	-1.6731041	-1.36639932	-1.50960537	1.60436346	1.50364419	-1.5917633	-1.00105598
augustus_masked-contig_376-processed-gene-0.8-mRNA-1	502.48	-9.49	-3.25	0	0	0	-2.12451	-1.87654228	-1.72135366	-1.47546324	1.63951248	1.53156403	-1.50716713	-1.06777059
augustus_masked-contig_207-processed-gene-0.22-mRNA-1	186.86	-16.93	-4.08	0	0	0	-2.22355	-2.15204012	-1.53396329	-1.76693513	1.69176415	1.6754868	-2.25123308	-1.80136863

Supplementary Figure 4

PHI-BLAST (log2 of fold change > 8

NRRL64463		Brown		Tan		Red							
**Differential Expression PHIBLAST**													
**LOG2 minus mean**								**qseqid**	**qlen**	**sseqid**	**PHI**	**Pathogeni**	**Reference**
**CP01**	**CP01-2**	**NPO22**	**NPO22-2**	**NPT22**	**NPT22-2**	**NPR22**	**NPR22-2**						
-4.22611	-4.43214	1.880779	1.854561	-2.58949	-2.0901	-3.35575	-3.28775	augustus_masked-contig_337-processed-gene-0.16-mRNA-1	747	R4I3H8	PHI:3075	PdChsVII	36651
-0.50189	-0.32417	1.501143	1.505236	-1.14335	-1.48735	-6.58127	-7.41309	snap_masked-contig_715-processed-gene-0.94-mRNA-1	6564	F8R4Y0	PHI:2311__PHI:	PKS__ACR	5530__559
0.505863	1.268305	1.365856	0.67013	-8.51373	-8.20945	-8.51373	-8.20945	augustus_masked-contig_69-processed-gene-0.10-mRNA-1	1656	Q86ZF1	PHI:261	ICL1	5022
-4.18982	-4.14975	1.333737	1.207805	0.202493	0.494109	-1.60486	-2.14975	augustus_masked-contig_85-processed-gene-0.25-mRNA-1	261	I1RIT1	PHI:11594	FgMFS1_(	5518
-0.04182	0.187627	1.296982	1.33787	-0.95936	-1.71049	-4.12928	-5.16993	augustus_masked-contig_658-processed-gene-0.29-mRNA-1	435	G4NDE1	PHI:1017__PHI:	ABC4	318829
-2.11548	-2.18982	1.206451	1.019629	0.230298	0.684645	-1.79355	-2.77479	augustus_masked-contig_580-processed-gene-0.13-mRNA-1	255	I1S0H5	PHI:7717	FgOXP1	5518
-0.017	0.101048	1.148435	1.11556	-0.55123	-0.6211	-3.15146	-3.17815	augustus_masked-contig_632-processed-gene-0.53-mRNA-1	675	A0A172QE	PHI:10412	SCD1	474922
-2.71234	-2.28517	1.082609	0.738288	0.332091	0.695143	-1.08707	-0.97824	augustus_masked-contig_191-processed-gene-0.43-mRNA-1	939	L7JF68	PHI:10457	Gls1	318829
-0.03612	0.149601	1.036858	0.840241	-0.4109	-0.10538	-2.17908	-2.5494	augustus_masked-contig_288-processed-gene-0.18-mRNA-1	924	I1S2J6	PHI:9039	Fgm2_(FG	5518
0.364092	0.457013	0.982449	0.893627	-0.46198	-0.40358	-6.49015	-6.21304	augustus_masked-contig_51-processed-gene-0.47-mRNA-1	918	Q12634	PHI:685__PHI:2	BUF__BUF	318829
-1.53138	-2.63421	0.976413	0.910114	0.754021	0.970656	-5.53138	-5.21917	augustus_masked-contig_377-processed-gene-0.9-mRNA-1	597	E9EN82	PHI:10684	MrHex1_(	568076
-1.96904	-1.98084	0.872713	0.733187	0.84796	0.99185	-3.13897	-3.3866	maker-contig_369-snap-gene-0.18-mRNA-1	1677	Q9P304	PHI:199	AOX1	5499
-0.73929	-0.35057	0.52059	0.852525	-0.28179	-0.71359	0.1939	-0.32145	augustus_masked-contig_93-processed-gene-0.95-mRNA-1	3543	Q4W9Q8	PHI:9255__PHI:	MsbA_(AF	746128
0.000143	0.265019	0.346574	0.27912	0.701199	0.593528	-3.28619	-3.71859	augustus_masked-contig_306-processed-gene-0.30-mRNA-1	1524	O93876	PHI:3987	Cpkk1	5116
-3.28862	-2.61321	0.267307	0.197632	0.891726	1.019845	-0.25376	-0.59499	augustus_masked-contig_730-processed-gene-0.26-mRNA-1	816	A0A384K1	PHI:10369	BcBGL5_(E	40559
0.119732	0.506212	0.226187	0.051953	0.253915	0.104636	-0.85912	-1.09605	augustus_masked-contig_59-processed-gene-0.59-mRNA-1	2523	Q870A3	PHI:315__PHI:7	PacC	5507
-0.32493	-0.13688	0.171188	-0.22571	0.591907	0.870601	-0.81492	-1.29719	augustus_masked-contig_409-processed-gene-0.11-mRNA-1	2091	Q4WZL4	PHI:2358__PHI:	PLD__Pld	746128
-3.53398	-2.93664	0.109878	0.539095	1.220909	1.192645	-0.98966	-2.93664	augustus_masked-contig_824-processed-gene-0.0-mRNA-1	372	Q5Y9C4	PHI:333	CaEcm33	5476
-0.76419	-0.35841	-0.00224	-0.01038	0.884134	0.848135	-0.81829	-1.22778	augustus_masked-contig_961-processed-gene-0.145-mRNA-1	2316	G4XKY8	PHI:2316	ChVel1	5016
0.360696	0.682042	-0.04601	-0.44788	0.71797	0.639278	-3.28658	-3.25263	augustus_masked-contig_569-processed-gene-0.51-mRNA-1	1737	I1RC73	PHI:1933__PHI:	GzZC248_	5518
-2.13141	-1.67463	-0.2475	0.074812	1.455621	1.35	-2.42146	-3.56648	augustus_masked-contig_781-processed-gene-0.11-mRNA-1	531	G4NAT7	PHI:3811	TDG4	318829
-3.27302	-2.86625	-0.30339	-1.20328	0.949374	0.983417	0.208108	0.537474	snap_masked-contig_926-processed-gene-0.7-mRNA-1	582	A0A384JTI	PHI:10366	BcBGL2_(E	40559
-1.27911	-1.15754	-0.5505	-0.71008	1.391781	1.454077	-1.83061	-2.31741	snap_masked-contig_394-processed-gene-0.122-mRNA-1	2346	E3QHX9	PHI:6227	KRE5	31870
-0.19751	0.33236	-1.16993	-1.19944	1.291492	1.109001	-2.08504	-2.75124	augustus_masked-contig_62-processed-gene-0.48-mRNA-1	717	I1RW14	PHI:10615	FgCrpA_(F	5518
1.888969	1.932886	-1.75489	-2.45943	-2.75489	-2.45943	-2.75489	-2.45943	augustus_masked-contig_818-processed-gene-0.0-mRNA-1	204	I1S097	PHI:4660	Chs3b	5518
-4.07948	-4.26679	-1.90956	-2.87447	1.868882	1.918089	-5.49452	-4.94486	augustus_masked-contig_17-processed-gene-0.20-mRNA-1	492	I1RPD3	PHI:6719	FgPLC1	5518
-1.77052	-1.30693	-1.95109	-1.99498	1.59323	1.635993	-1.21412	-2.07744	snap_masked-contig_267-processed-gene-0.13-mRNA-1	306	Q7Z8E8	PHI:419	CSH1	5476
-1.46053	-1.23469	-2.1383	-2.48358	1.311611	0.953406	-0.10888	0.545797	augustus_masked-contig_428-processed-gene-0.6-mRNA-1	4758	Q9C1I7	PHI:310	MgAtr4	1047171
-0.96322	-0.63108	-2.62271	-3.0543	0.646732	0.684893	0.814845	0.701632	augustus_masked-contig_520-processed-gene-0.3-mRNA-1	1689	G4MSF2	PHI:2985	MGG_045	318829
0.781307	0.830772	-2.80256	-3.92788	-1.67327	-1.41692	0.867453	0.832808	augustus_masked-contig_334-processed-gene-0.3-mRNA-1	210	Q4WT11	PHI:3323	SskB	746128
-2.30506	-2.22023	-4.33289	-4.44541	1.115853	1.12486	0.660651	0.640335	augustus_masked-contig_446-processed-gene-0.109-mRNA-1	363	B0BES1	PHI:1023__PHI:	BcnoxA__	40559
-0.68296	-1.10798	-4.4561	-5.43741	1.21057	1.574521	0.024693	-0.90343	augustus_masked-contig_602-processed-gene-0.66-mRNA-1	873	G2XDS1	PHI:8052	Vdmyo5	27337
-0.94342	-0.85798	0.400538	0.620066	-1.94342	-2.08037	0.925999	0.744055	augustus_masked-contig_17-processed-gene-0.13-mRNA-1	144	G4MXR2	PHI:8753__PHI:	MoChia1_	318829
-1.84389	-1.87591	-2.0191	-2.78238	0.577371	0.661065	0.987386	1.000681	augustus_masked-contig_72-processed-gene-0.41-mRNA-1	939	E9F082	PHI:2323	Mhk1	568076
-3.19549	-3.01451	0.692481	0.308829	-1.84402	-1.56282	0.997282	1.201044	augustus_masked-contig_329-processed-gene-0.12-mRNA-1	921	Q4WR83	PHI:2321	SidI	746128
-0.87235	-0.11746	-2.24159	-2.75489	0.104189	0.484578	1.115966	0.614346	augustus_masked-contig_955-processed-gene-0.0-mRNA-1	204	A0A0D1E1	PHI:11210	Cda7	5270
-3.05144	-3.001	-0.62791	-0.82605	0.05786	0.205658	1.131816	1.109563	snap_masked-contig_825-processed-gene-0.1-mRNA-1	1743	Q4WNT5	PHI:2534	ERG11A	746128
-2.20592	-2.26123	-2.68201	-3.35555	0.489176	0.858529	1.153019	0.911132	snap_masked-contig_228-processed-gene-0.23-mRNA-1	264	I1RBE8	PHI:9047	FgPah1_(F	5518
-2.11796	-1.66034	-1.65835	-2.44543	0.150205	-0.05969	1.228418	1.345155	snap_masked-contig_361-processed-gene-0.0-mRNA-1	1908	I1RIT1	PHI:11594	FgMFS1_(	5518
-2.59229	-2.40221	0.351126	-0.00335	-2.34245	-1.49604	1.239671	1.29785	augustus_masked-contig_726-processed-gene-0.76-mRNA-1	1023	O59939	PHI:222	PELB	474922
-3.06231	-2.89929	-0.67556	-1.00186	-0.34777	0.584177	1.303575	0.90105	augustus_masked-contig_663-processed-gene-0.58-mRNA-1	1629	F5HAA8	PHI:424	PCK1	5207
-2.25714	-2.48176	-1.35718	-2.38306	-0.37175	-0.16314	1.393759	1.452169	augustus_masked-contig_52-processed-gene-0.51-mRNA-1	1311	B0XTA5	PHI:2293	CycA	746128
-5.35462	-3.66297	0.115703	0.100801	-2.03269	-1.926	1.404717	1.370458	augustus_masked-contig_146-processed-gene-0.40-mRNA-1	879	P52496	PHI:219	LIG4	5476
-3.12963	-2.97824	-1.00486	-1.77503	-0.79803	-0.61332	1.491745	1.549531	augustus_masked-contig_243-processed-gene-0.27-mRNA-1	249	A0A0D2X0	PHI:11461	Mt1_(FOX	5507
-2.4144	-2.32367	-1.31244	-1.38854	-0.75242	-0.06213	1.493736	1.298918	snap_masked-contig_613-processed-gene-0.8-mRNA-1	1062	P87023	PHI:8716	KRE6	5476
-2.48839	-2.27098	-1.89627	-2.47981	-0.90187	-1.06753	1.593577	1.649113	augustus_masked-contig_93-processed-gene-0.93-mRNA-1	4311	Q9UW03	PHI:202__PHI:1	BcatrB	40559
-1.50623	-0.95842	-5.69209	-6.41785	-2.96963	-3.41785	1.807754	1.757073	snap_masked-contig_443-processed-gene-0.33-mRNA-1	600	G4NII5	PHI:2094	Yvc1	318829

Supplementary Figure 5

Melanin/Dothistromin/Aflatoxin Biosynthesis Pathways

		NRRL64463		Brown		Tan		Red			
**Dothistromin/Aflatoxin/Melanin related genes**		**LOG2 Minus Mean**									
**Name**	**Bonferroni**	**CP01**	**CP01-2**	**NPO22**	**NPO22-2**	**NPT22**	**NPT22-2**	**NPR22**	**NPR22-2**		
snap_masked-contig_715-processed-gene-0.94-mRNA-1	0	-0.50189	-0.32417	1.501143	1.505236	-1.14335	-1.48735	-6.58127	-7.41309	*pks1*	Non-reducing polyketide synthase (PKS1), **conidial Yellow pigment** (tetrahydroxynaphtalene synthase)
augustus_masked-contig_51-processed-gene-0.47-mRNA-1	0	0.364092	0.457013	0.982449	0.893627	-0.46198	-0.40358	-6.49015	-6.21304	*THNR*	**Elsinochrome reductase, photosensitive perylenequinone**, tetrahydroxynaphtalene reductase
augustus_masked-contig_435-processed-gene-0.16-mRNA-1	0	-1.55266	-1.59051	1.348543	1.274886	-0.08511	0.164312	-2.55759	-2.97123	*PL-C*	Phospholipase C-beta domain (related to elsinochr. Reduct??)
augustus_masked-contig_34-processed-gene-0.36-mRNA-1	0	-0.08576	0.005157	0.951841	0.803303	0.077748	0.253533	-3.87818	-4.07815	*Arp2*	(Versicolorin reductase BLAST NCBI general). BLAST toNCBI Fulvia is Hydroxynapthalene reductase-like protein Arp2. Translation and Motif search shown DHN, THN and TetraHN motifs. Melanin synthesis. Arp2 is part of the 6-gene cluster of A. fumigatus for DHN-melanin (Arp2: Apspergillus red-pink) makes T4HN to DHN by reduction
am-cont_632--0.53	0	-0.017	0.101048	1.148435	1.11556	-0.55123	-0.6211	-3.15146	-3.17815	*Scd1*	Scytalone dehydratase in Fulvia
am-cont_51_0.47	0	0.364091	0.457012	0.982449	0.893627	-0.46198	-0.40358	-6.49015	-6.21304	*dotA*	dotA, ***aflM***, *ver-1*, Versicolorin reductase, ketoreductase, **versicolorin A (VERA) reductase, necessary to con**
augustus_masked-contig_186-processed-gene-0.4-mRNA-1	0	-0.96069	-1.06625	1.53165	1.650975	-1.12152	-1.68265	-2.88455	-3.82685	AvnA	Averantin hydroxylase [Fulvia fulva]; Cytochrome P450 monooxygenase orf2 [Fulvia fulva]
augustus_masked-contig_291-processed-gene-0.10-mRNA-1	0	-2.28068	-2.00477	1.408109	1.501357	0.095466	-0.23986	-3.79788	-3.77774	*pksA*	*pksA*, ***aflC***, polyketide synthase, Dothistr pathway, conidial yellow pigment
augustus_masked-contig_469-processed-gene-0.3-mRNA-1	0	-2.63421	-2.28041	1.427827	1.418244	0.014866	-0.02041	-2.85563	-2.88244	*vbsA*	*vbsA*, ***aflK***, **versicolorin B synthase**
augustus_masked-contig_242-processed-gene-0.7-mRNA-1	0	-1.41101	-1.25133	1.267032	1.260321	0.116967	0.007075	-2.91223	-2.47768	*verB*	*verB*, ***aflL***, **versicolorin B desaturase**, oxidative desaturation of VERB to VERA
augustus_masked-contig_759-processed-gene-0.5-mRNA-1	0	-0.85212	-0.69165	0.936938	1.194847	0.037701	-0.35462	-0.98538	-1.69165	*DS30*	DS30 within Dothistr pathway
augustus_masked-contig_85-processed-gene-0.29-mRNA-1	0	-1.32807	-1.03156	1.04288	0.72627	0.24052	0.59247	-1.47416	-1.52020	*HAVN-del*	5'-**hydroxyaverantin dehydrogenase** [Fulvia] HAVN dehydrogenase
augustus_masked-contig_291-processed-gene-0.15-mRNA-1	0	-2.26796	-2.06828	1.347494	1.21578	0.165535	0.370025	-2.98739	-2.77085	*cypA*	*cypA*, ***aflV***, CytP450 monoxygenase, **Averufin monooxygenase** (cypX), AVR->HVN, Dothistr pathway
augustus_masked-contig_469-processed-gene-0.5-mRNA-1	0	-1.52573	-1.26567	1.188966	1.188522	0.273566	0.186663	-2.60813	-2.58402	*hexA*	*hexA*, ***aflA***, Fatty acid synthase, **fas-2**, Dothistr pathway
augustus_masked-contig_759-processed-gene-0.4-mRNA-1	0	-1.46084	-1.28977	1.227353	1.149804	0.191286	0.24405	-2.70305	-2.41248	*DS31*	DS31, translation elongation factor, within Dothistr pathway
snap_masked-contig_784-processed-gene-0.61-mRNA-1	0	-2.70116	-2.52723	1.331189	1.269434	0.147267	0.307602	-2.16707	-2.48835	*dotB*	dotB, oxidase
am-cont_242--0.6	0	-1.73674	-1.59579	1.141391	1.152403	0.379234	0.322596	-2.37059	-2.35297	*adhA*	alcohol dehydrogenase (AVN>HAVN>adhA to AVF); 5'-hydroxyaverantin dehydrogenase
am-cont_469--0.2	0	-1.90045	-1.68253	1.008961	0.832596	0.608639	0.75458	-2.3595	-2.18157	***hexB***	AvnA_p450 monoxygenase_AVN to HAVN then to AVF by adhA_Fatty acid synthase beta subunit hexB
augustus_masked-contig_469-processed-gene-0.1-mRNA-1	0	-1.14965	-0.82002	0.764511	0.643925	0.696021	0.703764	-2.11753	-2.04548	*hexB*	hexB, **fas-1**, fatty acid synthase beta subunit
augustus_masked-contig_1051-processed-gene-0.7-mRNA-1	0	-1.8691	-1.62436	1.033182	0.901867	0.594524	0.67175	-2.55853	-2.22325	*moxA*	*moxA*, ***aflW***, Dothistr pathway, Hydroxyversicolorone monooxygenase
augustus_masked-contig_242-processed-gene-0.9-mRNA-1	0	-1.22994	-1.036	0.612552	0.1601	0.851001	1.081993	-2.05319	-1.84685	*nor1*	norsolorinic acid ketoreductase **nor1** in Fulvia fulva
augustus_masked-contig_469-processed-gene-0.4-mRNA-1	0	-1.3354	-1.2511	1.0229	0.8510	0.4752	0.6312	-2.4606	-2.1372	*DS12*	DS12, Dothistromin pathway
augustus_masked-contig_678-processed-gene-0.11-mRNA-1	0	-0.56289	-0.26607	0.864867	0.824913	0.012303	0.063659	-1.01941	-1.50685	*DS33*	DS33, within Dothistr pathway
augustus_masked-contig_1053-processed-gene-0.29-mRNA-1	0	-0.72039	-0.39738	0.597492	0.58111	0.074481	0.037432	-0.27404	-0.47699	*AFAR*	*AFAR*, aflatoxin-aldehyde reductase, converts AFB1-dihydrodiol to AFB1-dialcohol (detoxification)
augustus_masked-contig_784-processed-gene-0.46-mRNA-1	0	-2.31601	-2.13143	0.779016	0.490424	0.891178	1.054708	-2.12956	-1.7878	*dotA*	dotA, ***aflM***, *ver-1*, Versicolorin reductase, ketoreductase, **versicolorin A (VERA) reductase, necessary to con**
augustus_masked-contig_784-processed-gene-0.33-mRNA-1	0	-1.03874	-0.96132	0.251676	0.087386	1.003703	1.117134	-1.6551	-1.97248	*dotD*	dotD, *aflC* thioesterase, Dothistr pathway
snap_masked-contig_291-processed-gene-0.25-mRNA-1	0	-1.83302	-1.69322	0.259254	0.079166	1.08526	1.178555	-1.31938	-1.43106	*avfA*	*avfA*, ***aflI***, oxidase Dothistr pathway, converts Averufin (AVR) to versiconal acetate (VHA)
augustus_masked-contig_784-processed-gene-0.47-mRNA-1	0	-1.82232	-1.74609	0.14294	-0.0248	1.107122	1.158037	-1.12366	-1.03682	*dotC*	dotC, ***aflT* toxin pump**, MFS transporter, Dothistr pathway
augustus_masked-contig_746-processed-gene-0.7-mRNA-1	0	-0.97924	-0.89988	0.859731	0.114035	0.507237	1.140906	-1.96168	-2.50125	*AvnA*	**Averantin hydroxylase** [Fulvia fulva]_Cytochrome P450 monooxygenase lcsl __AvnA_p450 monoxygenase_A
augustus_masked-contig_472-processed-gene-0.0-mRNA-1	0	-1.46479	-1.19621	0.25990	0.11983	1.10944	1.16156	-1.82268	-2.05900	aflR	aflR: Dothistromin biosynthesis regulatory protein aflR [Fulvia fulva]
augustus_masked-contig_472-processed-gene-0.8-mRNA-1	2.37E-10	-0.16882	-0.28418	0.17817	-0.12908	0.50889	0.89823	-0.84680	-1.31974	aflJ	aflJ: Dothistromin biosynthesis regulatory protein aflJ [Fulvia fulva] Esterase? VHA =>VAL before VerB
augustus_masked-contig_677-processed-gene-0.42-mRNA-1	0	-1.43566	-1.20269	-0.49873	-0.85081	0.84972	1.044973	0.164066	-0.07745	*avfA*	**Averufin oxidase A** [Fulvia fulva]
augustus_masked-contig_291-processed-gene-0.18-mRNA-1	0	-2.15508	-1.76518	-0.96283	-1.32157	0.88239	1.08948	0.50488	0.236021	*MFS*	putative MFS-type transporter, close to GlyHydro, not annotated in literature
augustus_masked-contig_243-processed-gene-0.37-mRNA-1	3x10-4	0.192645	-0.72792	-3.80735	-3.72792	1.050626	1.401363	-0.34792	-0.40599	aflX	putative Oxidoreductase **aflX**, putative amidase, averufin oxidase A
augustus_masked-contig_1051-processed-gene-0.6-mRNA-1	0	-3.52451	-3.08144	-0.71044	-0.72113	-0.49668	0.094234	1.374751	1.142569	*DS25*	DS25 within Dothistr pathway, amino acid permease
augustus_masked-contig_169-processed-gene-0.17-mRNA-1	0	-0.6607	-0.25605	-0.65831	-0.36981	-0.55909	-0.48603	1.039191	0.743948	*DS16*	DS16 within Dothistr pathway
snap_masked-contig_65-processed-gene-0.6-mRNA-1	0	-2.8468	-2.42371	1.201243	1.050644	0.471097	0.581121	-2.51081	-2.02234	*ordB*	
Dothistr	0	-1.33545	-1.25115	1.02288	0.85100	0.47516	0.63117	-2.46057	-2.13723	*hypC*	from polyketide to Norsolorinic Acid

CP01	NPO	NPT	NPR		
-	+	=	-	pksA	**main Dothistromin genes**
-	+	=	-	vbsA	functionally confirmed
-	+	+	-	dotA	
-	+	=	-	cypA	**Co-regulated with DS genes**
-	+	=	-	hexA	
-	+	+	-	moxA	
-	=	+	-	avfA	
-	+	=	-	dotB	**4 putative DS genes**
-	=	+	-	dotC	
-	=	+	-	dotD	
-	+	=	-	DS31	

Supplementary Figure 6

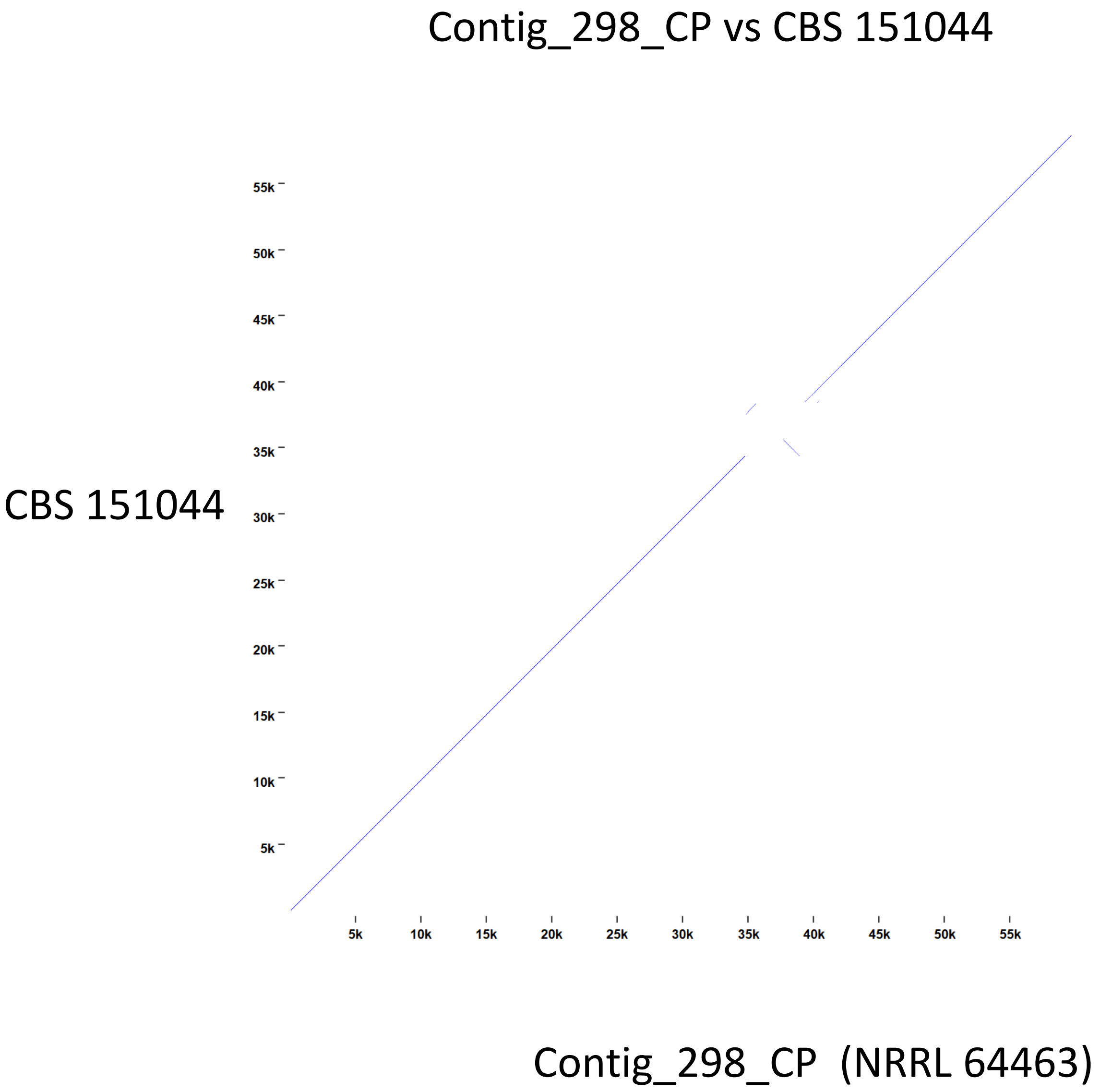

Supplementary Figure 7

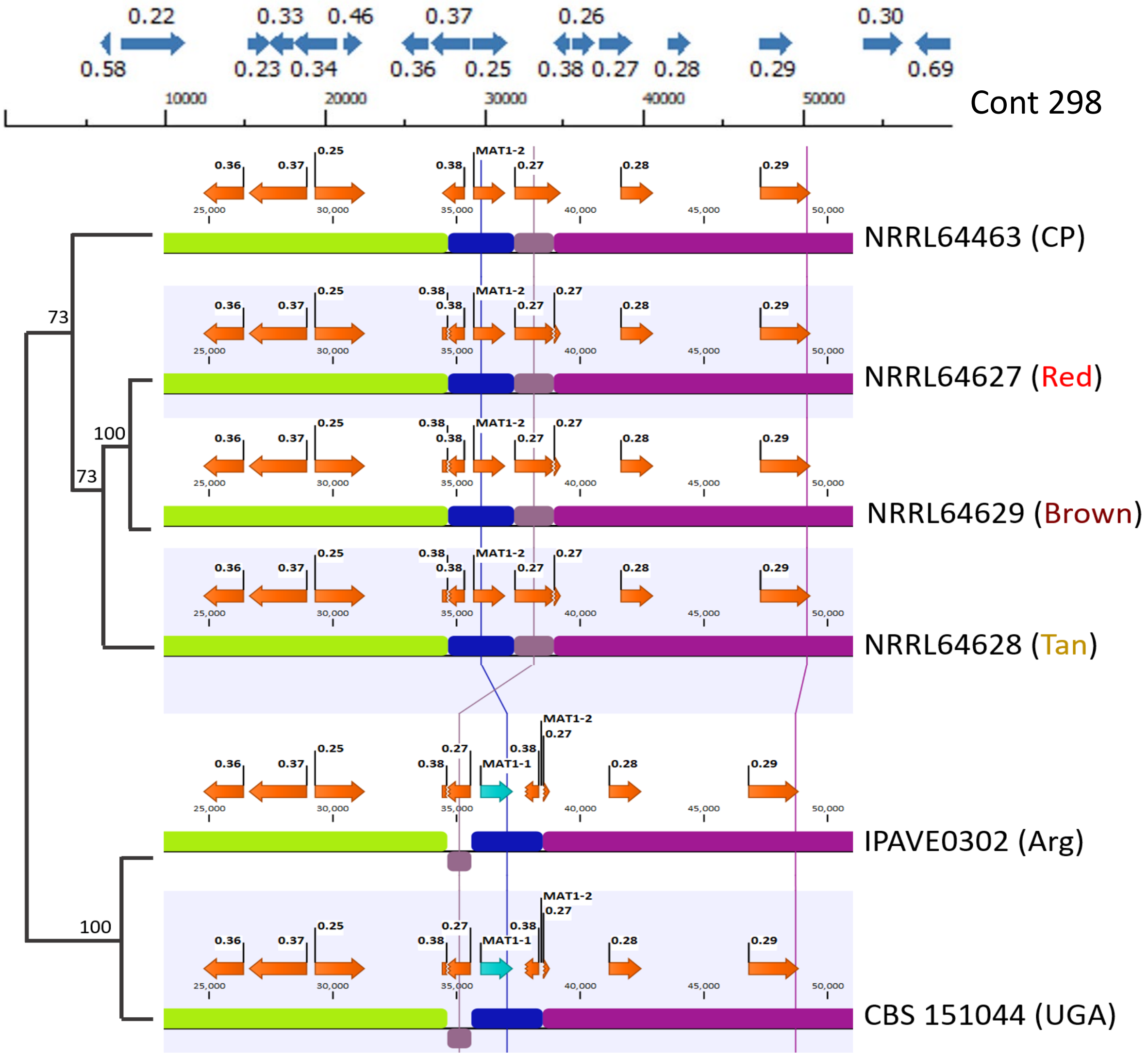

Supplementary Figure 8

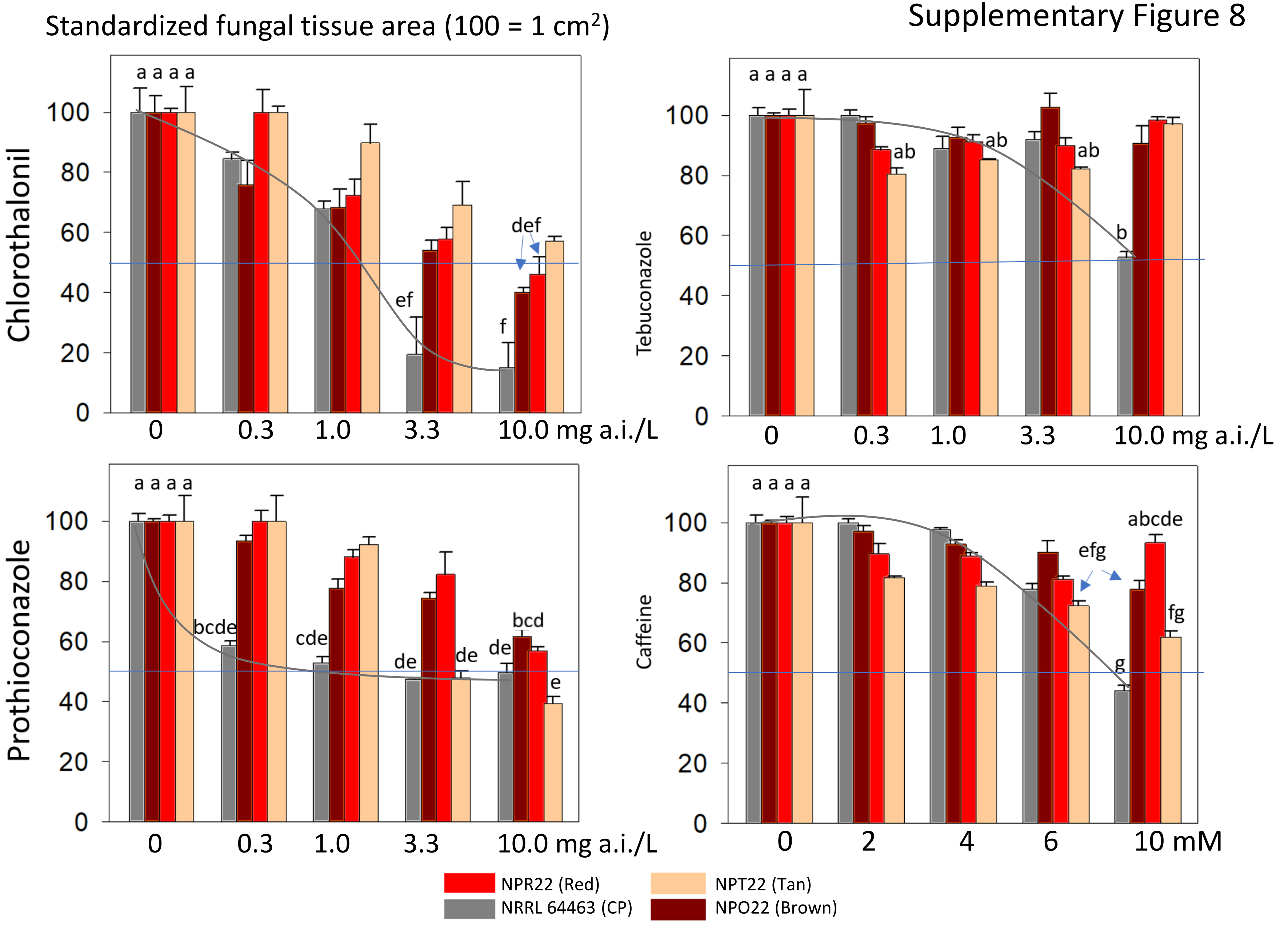

Supplement: Supplementary file 1 — Supplementary Material 1 [file 41598_2025_85953_MOESM1_ESM.pdf]
